# Integrative analysis the characterization of peroxiredoxins in pan-cancer

**DOI:** 10.1186/s12935-021-02064-x

**Published:** 2021-07-10

**Authors:** Lei Gao, Jialin Meng, Chuang Yue, Xingyu Wu, Quanxin Su, Hao Wu, Ze Zhang, Qinzhou Yu, Shenglin Gao, Song Fan, Li Zuo

**Affiliations:** 1 Department of Urology, Sir Run Run Shaw Hospital, Zhejiang University School of Medicine, Hangzhou, China; 2 Department of Urology, The First Affiliated Hospital of Anhui Medical University, Institute of Urology, Anhui Medical University, Anhui Province Key Laboratory of Genitourinary Diseases, Anhui Medical University, Hefei, China; 3 Department of Urology, The Affiliated Changzhou No. 2 People’s Hospital of Nanjing Medical University, Changzhou, China

**Keywords:** Peroxiredoxins, Pan-cancer, PRDX6, JAK2-STAT3, Bladder cancer

## Abstract

**Background:**

Peroxiredoxins (PRDXs) are an antioxidant enzymes protein family involved in several biological functions such as differentiation, cell growth. In addition, previous studies report that PRDXs play critical roles in the occurrence and development of carcinomas. However, few studies have conducted systematic analysis of PRDXs in cancers. Therefore, the present study sought to explore the molecular characteristics and potential clinical significance of PRDX family members in pan cancer and further validate the function of PRDX6 in bladder urothelial carcinoma (BLCA).

**Methods:**

A comprehensive analysis of PRDXs in 33 types of cancer was performed based on the TCGA database. This involved an analysis of mRNA expression profiles, genetic alterations, methylation, prognostic values, potential biological pathways and target drugs. Moreover, both the gain and loss of function strategies were used to assess the importance and mechanism of PRDX6 in the cell cycle of BLCA.

**Result:**

Analysis showed abnormal expression of PRDX1-6 in several types of cancer compared to normal tissues. Univariate Cox proportional hazard regression analysis showed that expression levels of PRDX1, PRDX4 and PRDX6 were mostly associated with poor survival of OS, DSS and PFI, and PRDX2 and PRDX3 with favorable survival. In addition, the expression of PRDX genes were positively correlated with CNV and negatively with methylation. Moreover, analysis based on PharmacoDB dataset showed that the augmented levels of PRDX1, PRDX3 and PRDX6 were significantly correlated with EGFR/VEGFR inhibitor drugs. Furthermore, knocking down of PRDX6 inhibited growth of cancer cells through the JAK2-STAT3 in bladder cell lines.

**Conclusions:**

PRDXs are potential biomarkers and therapeutic targets for several carcinomas, especially for BLCA. In addition, PRDX6 could regulate proliferation of cancer cell via JAK2-STAT3 pathway and involve into the process of cell cycle in BLCA.

**Supplementary Information:**

The online version contains supplementary material available at 10.1186/s12935-021-02064-x.

## Background

Peroxiredoxin (PRDX) belongs to a large group of antioxidant enzymes protein family which includes more than 3500 members [1]. Members of this family are characterized by a cysteine residue that is involved in reduction of peroxides [2], which are ubiquitously expressed in most organisms [3]. PRDXs can be classified into three subfamilies based on the number and location of the active cysteine residues and other factors. These subfamilies include: typical 2-Cys, atypical 2-Cys, and 1-Cys [4, 5]. Moreover, PRDXs can also be divided into six subfamilies based on the structural information around the active sites including AhpC-Prx1, BCP-PrxQ, Tpx, Prx5, Prx6, and AhpE [6]. Currently, there are 6 known mammalian PRDX members, namely PRDX1, PRDX2, PRDX3, PRDX4, PRDX5, PRDX6 [7]. The family members are involved in several biological processes, such as cell differentiation, metabolism [8], inflammation [9], cellular protection against reactive oxygen species (ROS) [10], embryonic development and cellular homeostasis [11].

Although the cardio- and neuroprotective effects have been fully established [12, 13], studies report that PRDXs play critical roles in the process of carcinogenesis and the development of drug resistance. Notably, PRDX1 was originally reported to act as an antioxidant enzyme because of its high susceptibility to oxidative stress [14], which plays important roles in cell growth, differentiation and apoptosis [15]. On the other hand, PRDX2 is associated with the proliferative, migratory and metastatic activities of melanoma [16]. Additionally, PRDX3 is highly expressed in tumor tissues of breast cancer, cervical cancer, liver cancer and prostate cancer [17–20]. Moreover, PRDX4 increases the proliferation rate of prostate cancer cell and promotes the metastasis of ovarian cancer, breast cancer, lung cancer and oral squamous cell carcinoma [21–24]. In addition, high expression of PRDX5 is associated with short overall survival (OS) in ovarian cancer [25]. Furthermore, high levels of PRDX6 have been reported with an invasive phenotype in breast cancer. Oncogenic role of PRDX6 was also reported in prostate cancer [26, 27]. Previous studies report that the augmented levels of PRDX1, PRDX3 and PRDX6 were associated with cisplatin resistance status for erythroleukemia, breast cancer and ovarian carcinoma [28, 29]. However, the comprehensive function of PRDXs in pan-cancer has not been explored.

In the present study, the mRNA expression profiles of PRDXs and their relationship with overall survival (OS), disease free interval (DFI), progression free interval (PFI), and disease specific survival (DSS) among 33 cancer types based on the TCGA database were explored. In addition, the association of expression levels of PRDXs with tumor microenvironment, genetic alteration and drug response activity were evaluated. ROS are critical mediators of tissue damage in patients with (BLCA) [30] are limited. Therefore, a comprehensive analysis of PRDXs was conducted. Moreover, effects of expression levels of PRDX6 in cell growth, apoptosis and the potential mechanisms were explored in T24 and TCCSUP cells.

## Materials and methods

### TCGA pan-cancer data

RNA-seq, clinical information, stemness scores based on mRNA (mRNAsi) and immune subtypes data for 33 cancer types were obtained from Xena browser (https://xenabrowser.net/datapages/). Out of the samples, only 18 cancer types had adjacent normal tissue samples. These samples were used to explore gene expression profiles in tumors compared with normal tissues. In addition, in order to analyze the correlation between gene expression (as continuous variable) and patient prognosis, patients with corresponding survival information were included. The OS, DFI, PFI and DSS data of PRDXs were analyzed based on HIPLOT dataset (https://hiplot.com.cn/advance/ucsc-xena-shiny).

### Tumor immune subtypes analysis

Six immune subtypes were used to explore immune infiltration in tumor environment [31]. Immune subtype was used to determine the relationship between PRDXs expression and immune infiltrate types in tumor microenvironment using ANOVA methods. Tumor stemness data obtained from TCGA dataset were performed to assess the stem-cell-like levels of tumor cells [32]. The association between cancer stemness and PRDXs expression profiles was examined using Spearman correlation method.

### Genetic alteration and DNA methylation

Genetic alterations, including mutation and copy number variation (CNV) data of different cancer types were retrieved from cBioPortal (http://www.cbioportal.org/). The association of CNV, methylation and tumor types were analyzed using GSCALite tool (http://bioinfo.life.hust.edu.cn/web/GSCALite/). Correlation analyses between mRNA expression levels of PRDXs and CNV levels, and methylation in BLCA were released from cBioPortal. Data on methylation levels between tumor and normal tissues in BLCA were obtained from UALCAN (http://ualcan.path.uab.edu).

### Drug and pathway analysis

Global proportion plot and heatmap of PRDXs genes in 10 tumor-associated pathways were generated using GSCALite (http://bioinfo.life.hust.edu.cn/web/GSCALite/). Data on PRDXs-related drugs were from PharmacoDB (https://pharmacodb.pmgenomics.ca/). The cut-off was absolute value of correlation more than 0.1 and *P* < 0.05 (Additional file 1: Table S3).

### Verification of expression profiles and pathway analyses of PRDXs

The Human Protein Atlas (HPA) database (http://www.proteinatlas.org/) was used to verify the protein level of PRDXs. Prognostic value of the PRDXs was verified by using survival (https://github.com/therneau/survival) and survimer (https://github.com/kassambara/survminer/) package. GSEA (http://software.broadinstitute.org/gsea/index.jsp) package was applied to analyze the differential pathways based on KEGG gene sets.

### Cell culture and lentiviral of PRDX6, STAT3

T24, TCCSUP and HEK293T cell lines were purchased from the American Type Culture Collection company (ATCC, Manassas, VA). Cells were cultured in DMEM media and maintained in a humidified 5% CO2 environment at 37 °C [33]. Lentiviral pLKO-vec, pLKO-shPRDX6 were transfected into 293T cells and harvested according to our previous paper [34]. For shPRDX6 knocking-down stable expression cell model, puromycin (2 μg/ml) was added to select the stably transduced cells. The lentiviral soups were stored in − 80 ℃ for further use. The constitutively active oeSTAT3 lentiviral vector (plasmid #24983) was purchased from Addgene (Cambridge, MA, USA).

### Western blot assay

Cells were lysed, quantified and equal 30 µg protein, was separated on 10% SDS/PAGE gel, and then transferred onto PVDF membranes (Millipore, Billerica, MA). After blocking the membrane for 2 h with 5% BSA at room temperature, then the membrane was incubated in primary antibodies at 4℃ for overnight. Next, the membrane was rinsed 3 times and incubated with secondary antibodies for 1 h at room temperature. Finally, the protein bands imaged by ECL system (Thermo Fisher Scientific). The primary antibodies used in the study for western blot were as follow: β-actin (Santa Cruz, #sc-8432, CA), PRDX6 (Abcam, #ab59543, USA), CDK4 (Santa Cruz, #sc-56277, CA), CDK6 (Santa Cruz, #sc-39049, CA), BCL-2 (Santa Cruz, #sc-20067, CA), JAK2 (Santa Cruz, #sc-390539, CA), p-STAT3 (Santa Cruz, #sc-8059, CA), STAT3 (Santa Cruz, #sc-8019, CA).

### Cell viability assay, Colony formation assay and cell cycle analysis

Stably expressed cells (5 × 10^3^/well) were seeded in 96-well plates. Cell viability was measured using Cell Counting Kit-8 (CCK-8, MedChemExpress, HY-K0301) method. Cells were plated into 6-well plates at a density of 1 × 10^3^ cells per well. After 14 days. colonies were rinsed twice with PBS, fixed with 100% methanol, and stained with 0.1% crystal violet, then the cell numbers were calculated. For cell cycle assay, cells were harvested and washed with cold PBS, and fixed in 70% cold ethanol. Cells were stained with 50 mg/L propidium iodide for 30 min, before analysis with a fluorescence-activated cell sorter (BD FACS Flow Cytometer, 342975).

### Statistical analysis

The R-3.6.2 software was performed for statistical analyse and generation of images. Comparison of PRDXs mRNA expression between tumor and normal tissues were performed using Wilcox signed- rank test. Comparison of multi-groups was performed using one way ANOVA (Analysis of variance) methods, then LSD test (Fisher's Least Significant Difference test) for post-hoc correction. Spearman's correlation analysis was utilized to explore the correlation between continuous variables. Univariate cox proportional hazard regression models or Log-rank tests were used to assess the correlations between gene expression and patient overall survival. *P* value < 0.05 was set as statistically significant. Each statistical test was two-sided.

## Results

### The gene expression and survival analysis of PRDXs in pan-cancer

**Fig. 1:**
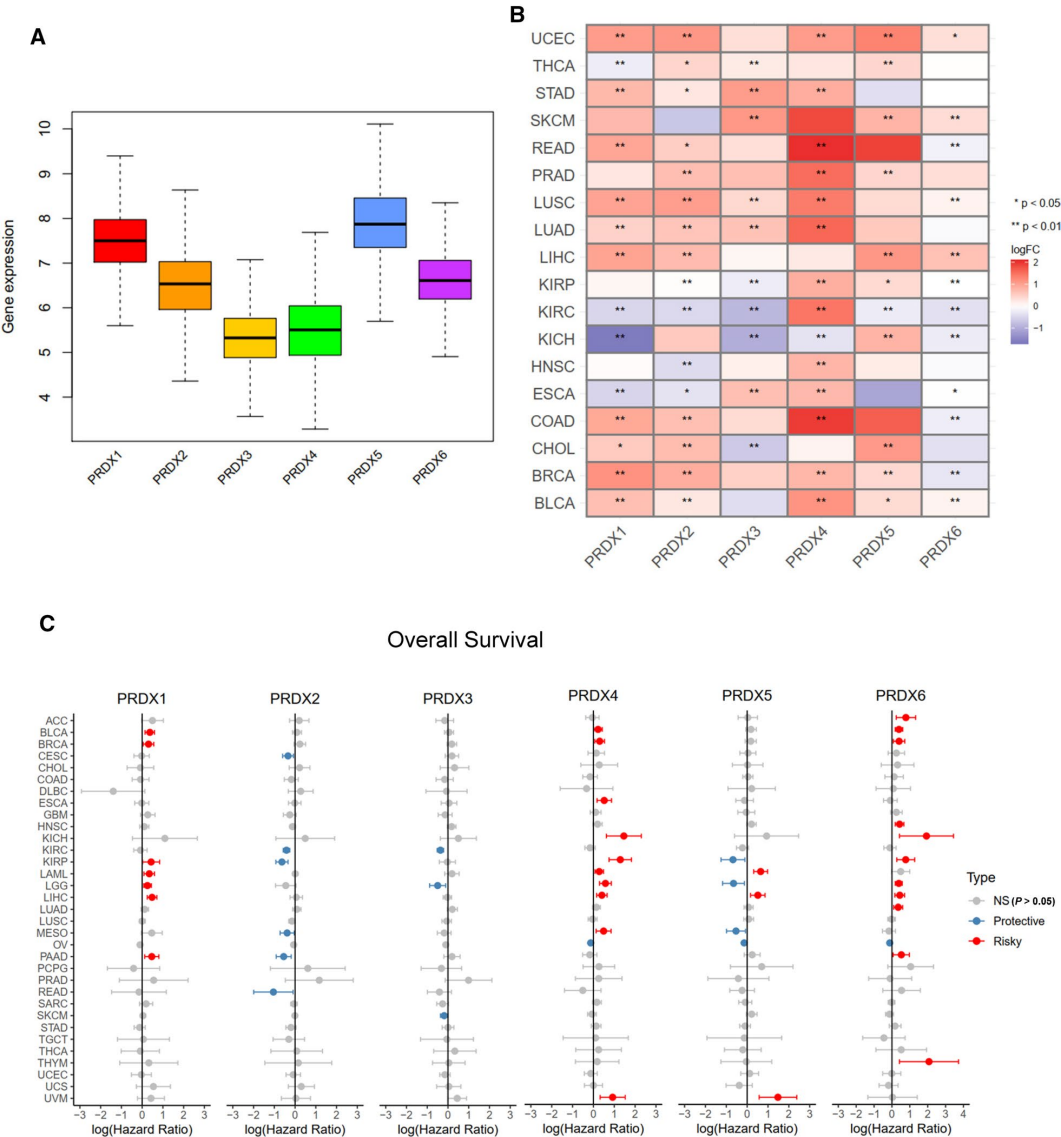
Expression levels of PRDX genes and association with overall survival in pan-cancer. **A** Boxplot to display the distribution of PRDX genes expression across all 33 type tumors. **B** Heatmap to show the difference of PRDX gene expression comparing tumor to normal samples based on log2(fold change) for 18 tumor types. **C** The forest plots for overall survival with hazard ratios (log10) and 95% confidence intervals for 33 different cancer types

The clinical information of 33 tumors and corresponding cancer tissue RNA-seq data were retrieved from the TCGA database. These data were used to explore expression patterns and prognostic value of PRDXs in different types of cancer. Analysis showed that significant differences in expression patterns of six PRDX genes across different tumor types (Fig. 1A). Notably, when compared to adjacent or normal tissues, PRDX1, PRDX2, PRDX4 and PRDX5 were significantly up-regulated expression in several cancer types (UCEC, READ, BLCA, BRCA, CHOL, COAD, LIHC, THCA), while PRDX3 and PRDX6 were down-regulated expression in kidney cancers (Fig. 1B, Additional file 1: Figure S1). Univariate Cox proportional hazard regression analysis showed that expression of PRDX1, PRDX4 and PRDX6 were mostly associated with poor survival of OS, DSS and PFI, and PRDX2 and PRDX3 may be protective factors (Fig. 1C, Additional file 1: Figure S2), such as PRDX1, PRDX4 and PRDX6 were risky factors for BLCA of OS, DSS and PFI, while PRDX2 and PRDX3 were protective factors for KIRC of OS, LGG of DSS, KIRP and STAD of PFI (Additional file 1: Figure S2).

### Genetic alteration and methylation of PRDXs in pan-cancer

**Fig. 2:**
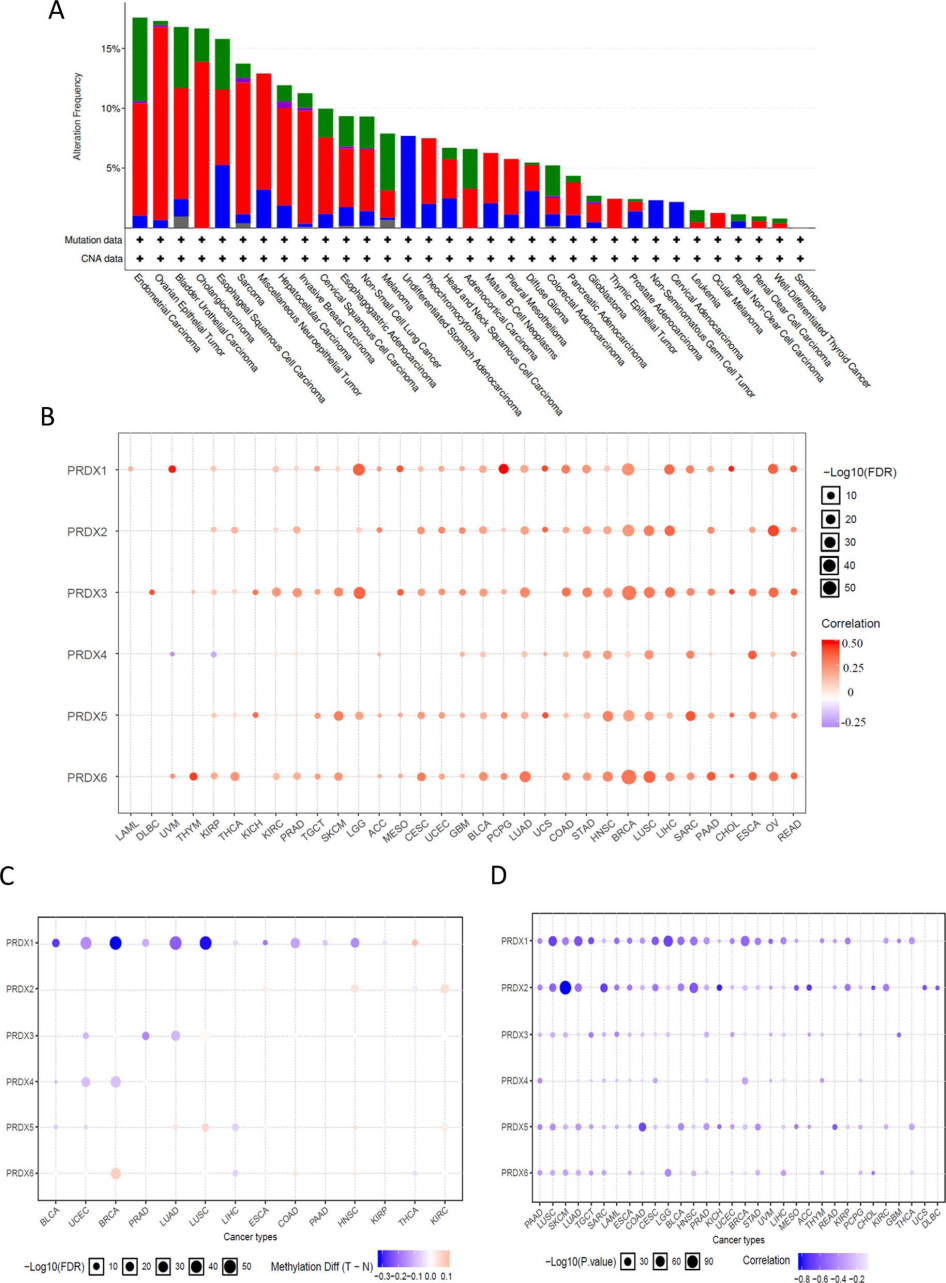
The genetic alterations of PRDXs and associations with mRNA expression. **A** Total genetic alterations of PRDX genes based on cBioportal website. **B** Bubble map indicated the relationship between PRDX mRNA expression and CNV. Red dots indicate that gene CNV levels were positively associated with mRNA expression, blue dots indicate the negative association. **C** The differential methylation of PRDXs between TCGA cancer and normal tissues. Blue dots show methylation down-regulation in tumors, and red dots show methylation up-regulation in tumors. **D** Associations between DNA methylation and PRDXs expression

Genetic alterations of PRDXs in the different cancers were collected from cBioprotal website. The top-five tumors with PRDXs genetic alterations were endometrial carcinoma, ovarian epithelial tumor, bladder urothelial carcinoma, cholangiocarcinoma, Esophageal squamous cell carcinoma (Fig. 2A). The mutation distributions of each PRDX were presented in Additional file 1: Figure S3. Then the copy number variation (CNV) and methylations of PRDXs in pan-cancer were explored based on GSCA Lite. The results demonstrated that the mRNA expressions of PRDXs was mainly positively correlated with CNV (Fig. 2B), while negatively with methylation (Fig. 2C, D). This finding implies that PRDXs might be the CNV and methylation drive genes.

### Associations between PRDXs and immune subtypes, tumor stemness, drug sensitivity and signaling pathways

**Fig. 3:**
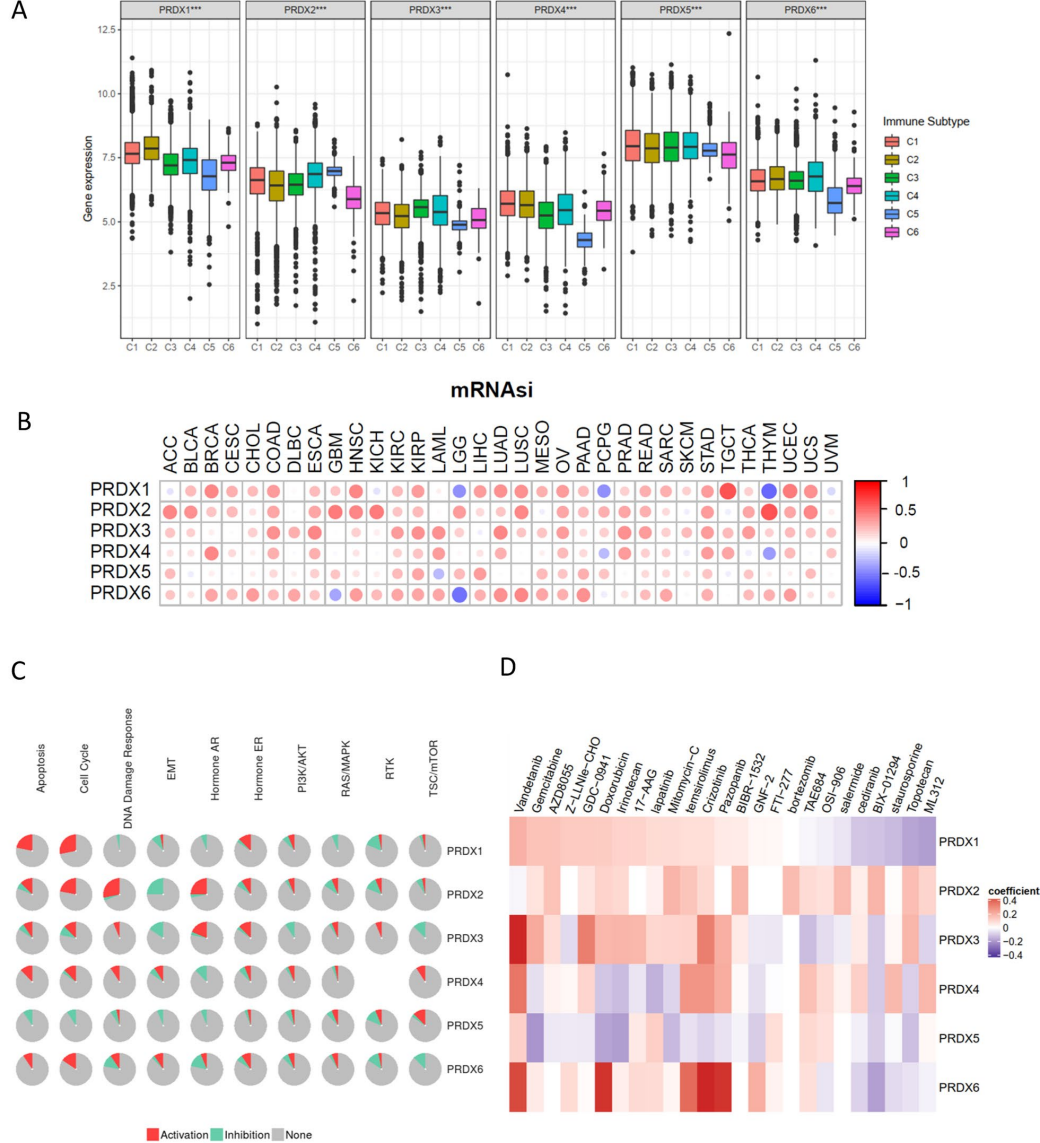
The immune infiltrate subtypes, cancer stemness, pathways and sensitivity to drugs of PRDXs. **A** Association of PRDX gene expression with immune infiltrate subtypes among all the tumor types tested by ANOVA. **B** Correlation plots of the relationship between PRDX gene expression and tumor stemness of 33 diverse tumor types. **C** Fan diagrams show the percentage distribution of PRDXs’ function (activation or inhibition) for related pathway in all cancers. **D** Heatmap of drug sensitivity/tolerance of PRDXs with high expression

Six subtypes of immune infiltrates, C1 (wound healing), C2 (INF-r dominant), C3 (inflammatory), C4 (lymphocyte depleted), C5 (immunologically quiet), and C6 (TGFβ dominant) were obtained from the study of David et al. [35]. Correlations between higher levels of PRDX1, PRDX4, PRDX6 and type 1, 2, and 6 infiltrates (C1, C2 and C6), suggesting the tumor promoting effect of these PRDXs (Fig. 3A). On the contrary, PRDX2 and PRDX3 showed higher expression levels in C4 subtypes, indicating that they are involved in tumor suppression. PRDXs have been previously reported to play a role in drug resistance and drug resistance is closely related to tumor stemness [32], therefore, the relationship between PRDXs expression profiles and tumor stemness were analyzed. Correlation analysis showed that PRDXs were strongly associated with the tumor stemness across different cancer types (Fig. 3B, Additional file 1: Table S1). Moreover, the correlation between tumor stemness and TGCT was significantly high with a correlation coefficient to 0.66 (*P* < 0.001). Then the relationship between PRDXs and 10 cancer-related pathways were analyzed based on GSCALite website (Fig. 3C). The results showed that PRDX1 was significantly associated with the activation effect of apoptosis, cell cycle, and inhibition of EMT and RTK. Furthermore, PRDX2 showed significant correlation with activation effect of apoptosis, cell cycle, DNA damage response and hormone AR, while inhibition of EMT and RTK. PRDX1, PRDX2, PRDX3, PRDX4, and PRDX6 were associated with the activation effect of apoptosis, cell cycle, while PRDX5 significantly associated with inhibition of apoptosis and cell cycle. The top five cancer drugs potentially associated with each of the PRDX members (*P* < 0.05, standardized coefficient > 0.1) were analyzed based on PharmacoDB [36] database (Fig. 3D, Additional file 1: Table S3). The results demonstrated that pazopanib, vandetanib, lapatinib and cediranib were associated with PRDX1, PRDX3, PRDX4, PRDX6, which suggested that the PRDXs may be strongly associated with the EGF and VEGF signaling pathway. Moreover, Gemcitabine, Doxorubicin, AZD8055 (mTOR inhibitor), GDC-0941 (PI3K inhibitor), Irinotecan (cytotoxic chemotherapy drug), 17-AAG (HSP90 inhibitor), Mitomycin-C (cytotoxic), and temsirolimus drugs were significantly associated with PRDXs (Fig. 3D).

### Expression profiles, genetic alteration of PRDXs and the correlations with immune subtypes, stage in bladder cancer

**Fig. 4:**
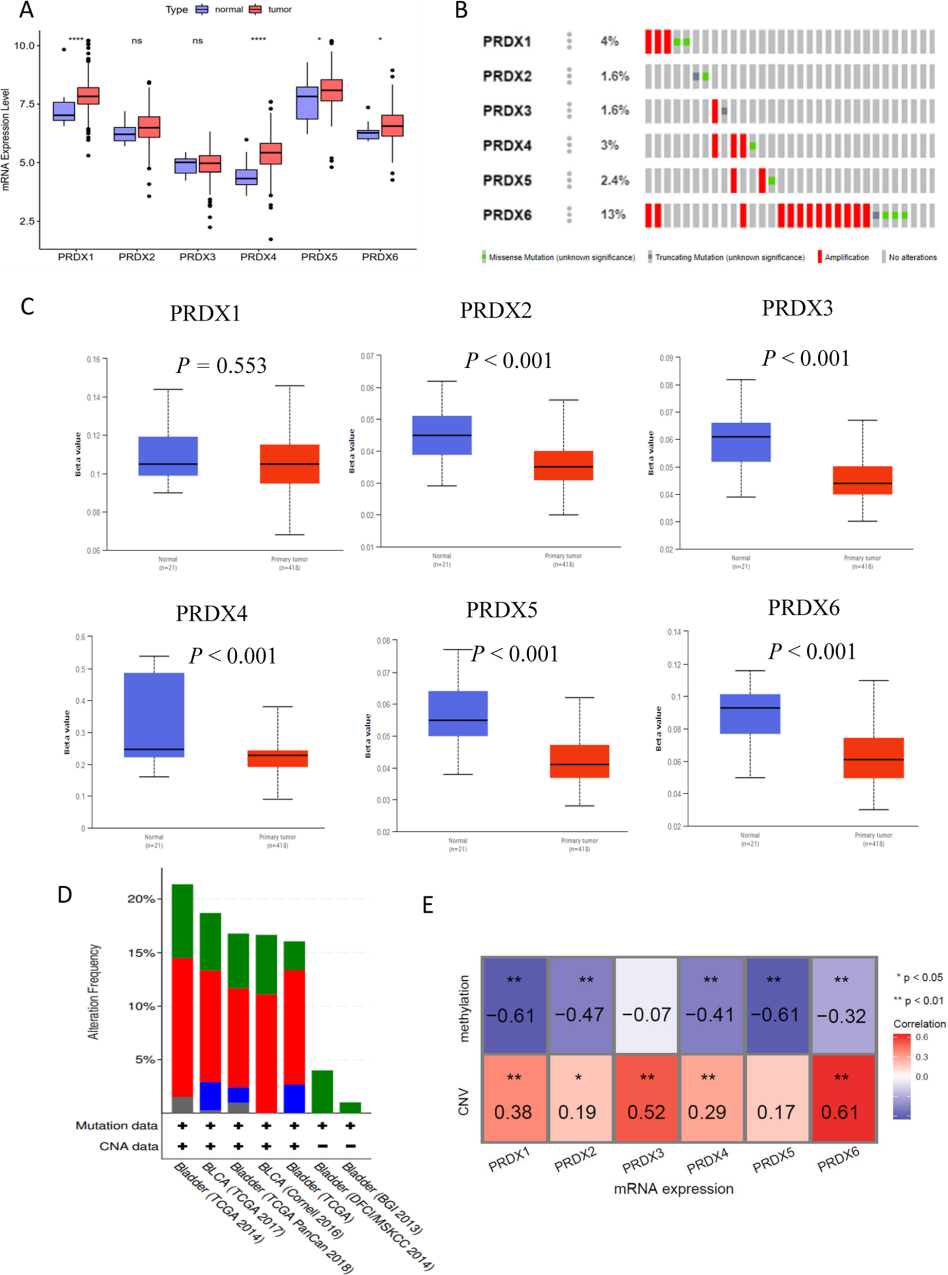
Expression profiles and genetic alterations of PRDXs in bladder cancer based on TCGA dataset. **A** The mRNA expression profiles of PRDXs in tumor compared to normal tissues. **B** The genetic alterations of PRDXs in bladder cancer. **C** The mRNA methylation levels of PRDXs in tumor compared to normal tissues. **D** The global percentage shows genetic alterations of PRDXs in different bladder cancer cohorts based on cBioprotal website. **E** Heatmap of the associations between PRDXs mRNA expression and CNV levels, DNA methylation in bladder cancer

**Fig. 5:**
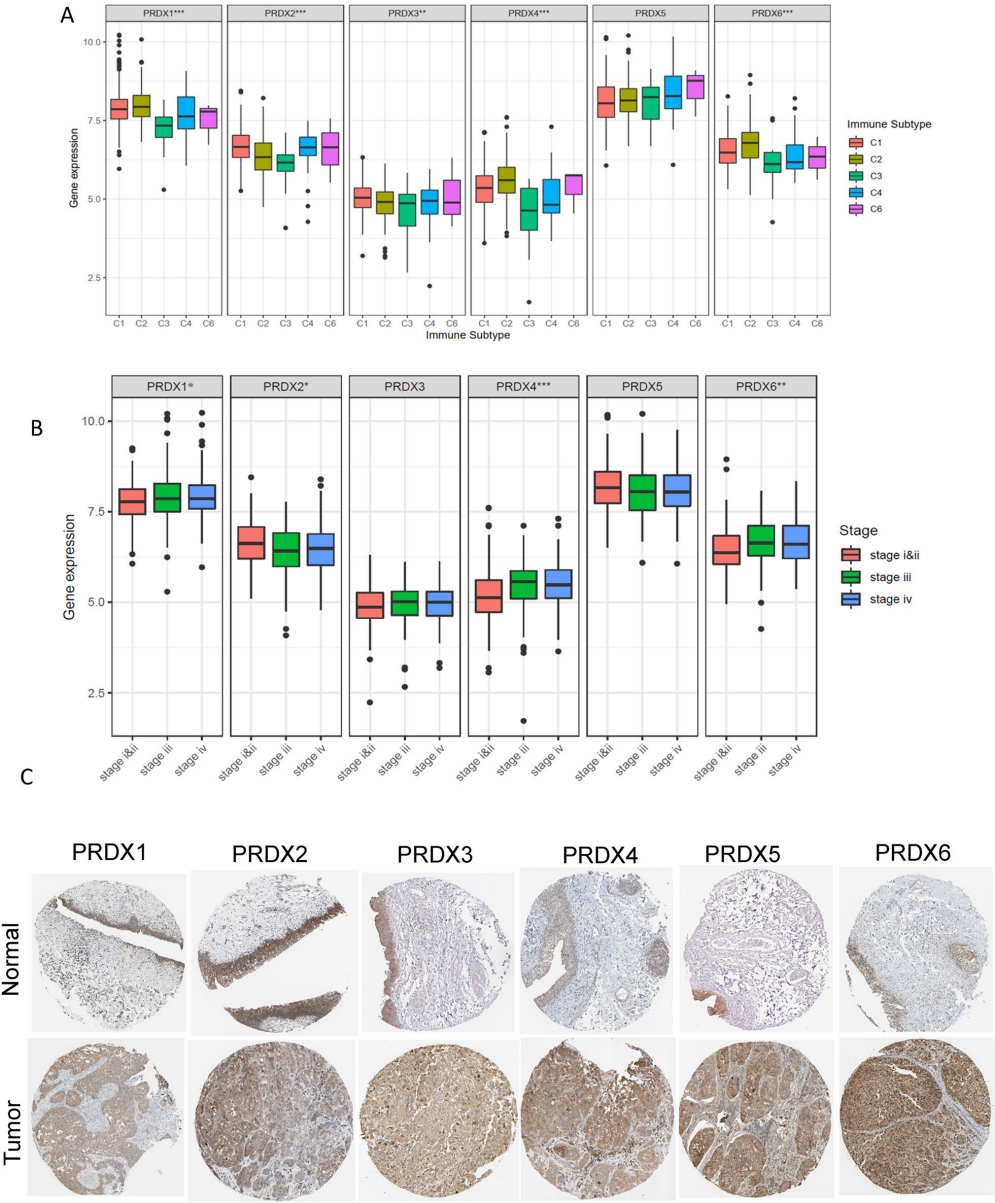
The PRDXs mRNA expression of different immune infiltrate subtypes, stage in bladder cancer and protein expression of PRDXs in tumor and normal bladder tissues. **A** Associations of PRDX gene expression with immune infiltrate subtypes. **B** Associations of PRDX gene expression with different stage. **C** Immunohistochemistry images obtained from the HPA database

ROS is associated with tissue damage in BLCA patients, therefore, a comprehensive analysis of the role of PRDXs in BLCA was performed. The mRNA expression levels and genetic alterations were analyzed. All PRDXs were highly expressed in BLCA tumor tissues compared with normal tissues (Fig. 4A), though there is no significant different in expression levels of PRDX2, PRDX3 between tumor and normal tissues. Genetic alterations of PRDX1-6 were 4%, 1.6%, 1.6%, 3%, 2.4%, 13%, respectively (Fig. 4B, D), mainly CNV mutation (Fig. 4E). The methylation levels of PRDX2-6 were significantly low in tumor when compared with normal tissues (*P* < 0.001) (Fig. 4C). Moreover, the mRNA expression levels of PRDXs were significantly correlated with CNV and methylation in tumor tissues (Fig. 4E). These findings were in consistence with the analyses of pan-cancer. In addition, analysis of immune subtypes showed that high expression levels of PRDX1, PRDX4 and PRDX6 were significantly correlated with type 1, 2 infiltrates (C1, C2). On the other hand, low expression levels of PRDX1, PRDX4 and PRDX6 were correlated with C3 subtypes, indicating that they are potential tumor promoters, similar to pan-cancer results (Fig. 5A). Furthermore, the mRNA expression levels of PRDX1, PRDX4 and PRDX6 were closely related with tumor stage of BLCA (Fig. 5B, Additional file 1: Figure S4B). The immunohistochemistry (IHC) results from the Human Protein Atlas (HPA) database showed high expression levels of PRDXs in BLCA (Fig. 5C).

### The diagnostic, prognostic value and GSEA results of PRDXs in bladder cancer

**Fig. 6:**
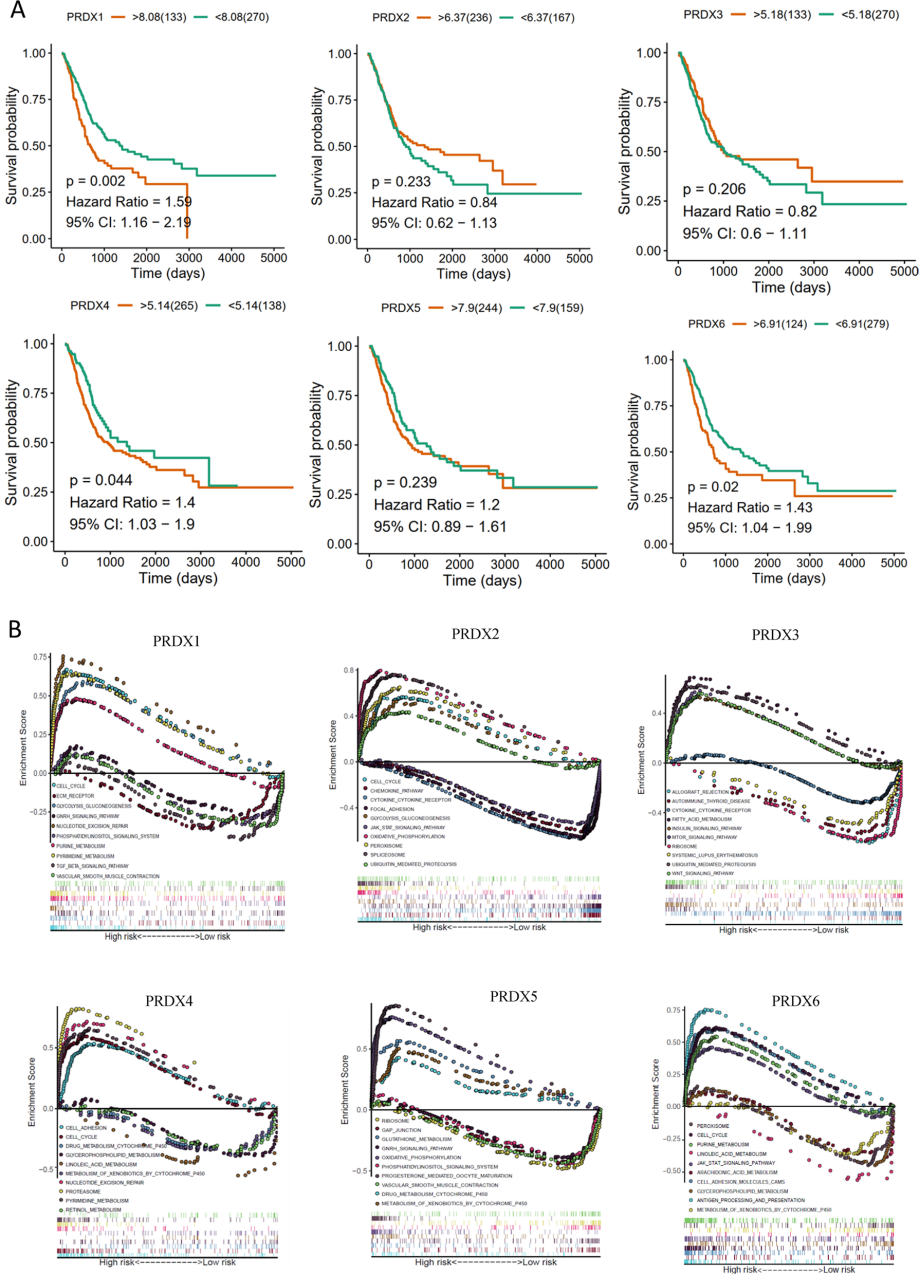
The overall survival and GSEA plots of RPDXs in bladder cancer based on TCGA. **A** The overall survival curves of PRDX genes. **B** The KEGG enrichment results of PRDX genes using GSEA method

In order to explore the diagnostic of PRDXs in BLCA, the area under the receiver operating curve (AUC) was calculated. The Kaplan Meier (KM) plot and Log-rank tests were performed to determine the relationship between PRDXs and OS. The results showed that the AUC of PRDXs were 0.799, 0.63, 0.452, 0.837,0.66 and 0.673 (Additional file 1: Figure S4A). The KM results demonstrated that PRDX1 (*P* = 0.002), PRDX4 (*P* = 0.044) and PRDX6 (*P* = 0.02) were associated with poor survival (Fig. 6A). Moreover, the univariate Cox results showed that PRDX1 (*P* = 0.001) and PRDX6 (*P* = 0.004) were prognostic factors, while only PRDX6 (*P* = 0.071) may be the independent prognostic factors based on the multivariate Cox results (Additional file 1: Table S2). Subsequently, Gene set enrichment analysis (GSEA) methods was utilized to explore the potential biological pathways in BLCA between low and high expression RPDX groups. The significant pathways followed the criterion (FDR < 0.05) in enrichment of KEGG biological terms from MSigDB Collection were presented in Fig. 6B. The results demonstrated that PRDX1, PRDX2, PRDX4 and PRDX6 were associated with cell cycle, and cell adhesion pathways (such as ECM receptor, focal adhesion, GAP junction, and cell adhesion molecules CAMs). PRDX1 were strongly associated with TGF-beta pathway, while PRDX2, PRDX6 with JAK-STAT pathway, PRDX3 with mTOR pathway. All PRDXs were significantly associated with cell metabolism processes, such as glycolysis gluconeogenesis, purine metabolism, fatty acid metabolism, drug metabolism cytochrome P450, retinol metabolism and metabolism of xenobiotics by cytochrome P450 (Additional file 1: Fig. 6B).

### Knockdown of PRDX6 inhibits the cell proliferation of BLCA

**Fig. 7:**
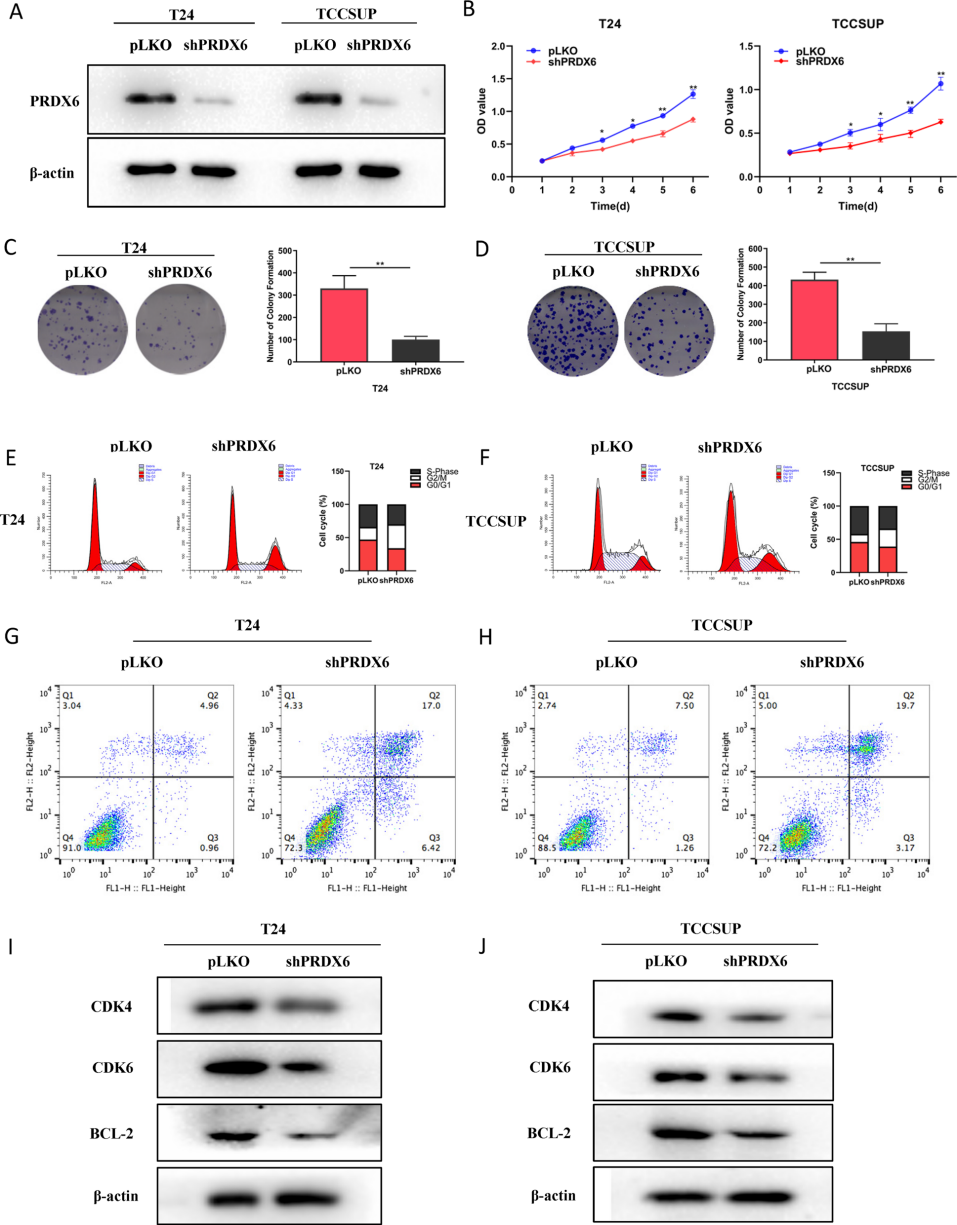
Knockdown of PRDX6 inhibits the cell proliferation of BLCA. **A** Knockdown efficiency of PRDX6 in T24 cells and TCCSUP cells. **B** CCK8 assay revealed cell proliferation in T24 cells and TCCSUP cells. **C**, **D** PRDX6 depletion decreased colony forming ability of T24 cells (**C**) and TCCSUP cells (**D**). **E**, **F** Knockdown PRDX6 arrested cell population in G2/M-phase both in T24 cells (**E**) and TCCSUP cells (**F**) by flow cytometer. **G**, **H** Knockdown PRDX6 increase the apoptosis of T24 cells (**G**) and TCCSUP cells (**H**). **I**, **J** Knockdown PRDX6 decrease the protein level of CDK4, CDK6 and BCL2. *P < 0.05, **P < 0.01

As PRDX6 had high expression in tumor tissues with poor prognosis, and high genetic alterations rate in BLCA, then PRDX6 was selected to explore its roles in BLCA. We first knocked down PRDX6 in T24 and TCCSUP cell lines (Fig. 7A), then tested whether PRDX6 had any biological functions in BLCA cells. CCK8 assay was used to detect the effect of PRDX6 on cell proliferation in BLCA cells. The results revealed that knockdown of PRDX6 in T24 and TCCSUP cells significantly suppressed the cell growth (Fig. 7B). Similarly, colony formation assay showed that knockdown of PRDX6 in T24 and TCCSUP cells reduced the colony number compared with the corresponding controls (Fig. 7C, D). Next, FACS analysis was used to investigate the mechanism of PRDX6 in modulating progression of cell cycle. The results showed that PRDX6 deletion resulted in an increased percentage cells in G2/M phase and a decreased percentage of cells in the S phase in T24 and TCCSUP cells (Fig. 7E, F). The Annexin V-APC and 7-AAD staining was performed to assess the role of PRDX6 in apoptosis. The scatter plots demonstrated a higher apoptotic index both in the T24 and TCCSUP cells with shPRDX6 (Fig. 7G, H).

Our previous analysis showed that PRDX6 was highly correlated with cell cycle. Therefore, the core genes, CDK4, CDK6 and BCL2 were selected to detect. The result showed that knockdown PRDX6 significantly decreased the protein levels of CDK4, CDK6 and BCL-2 in the T24 cells (Fig. 7I) and TCCSUP cells (Fig. 7J). In summary, these results demonstrated that knockdown of PRDX6 inhibits the cell proliferation of BLCA.

### PRDX6 promotes bladder cancer cell proliferation via JAK2-STAT3 pathway

**Fig. 8:**
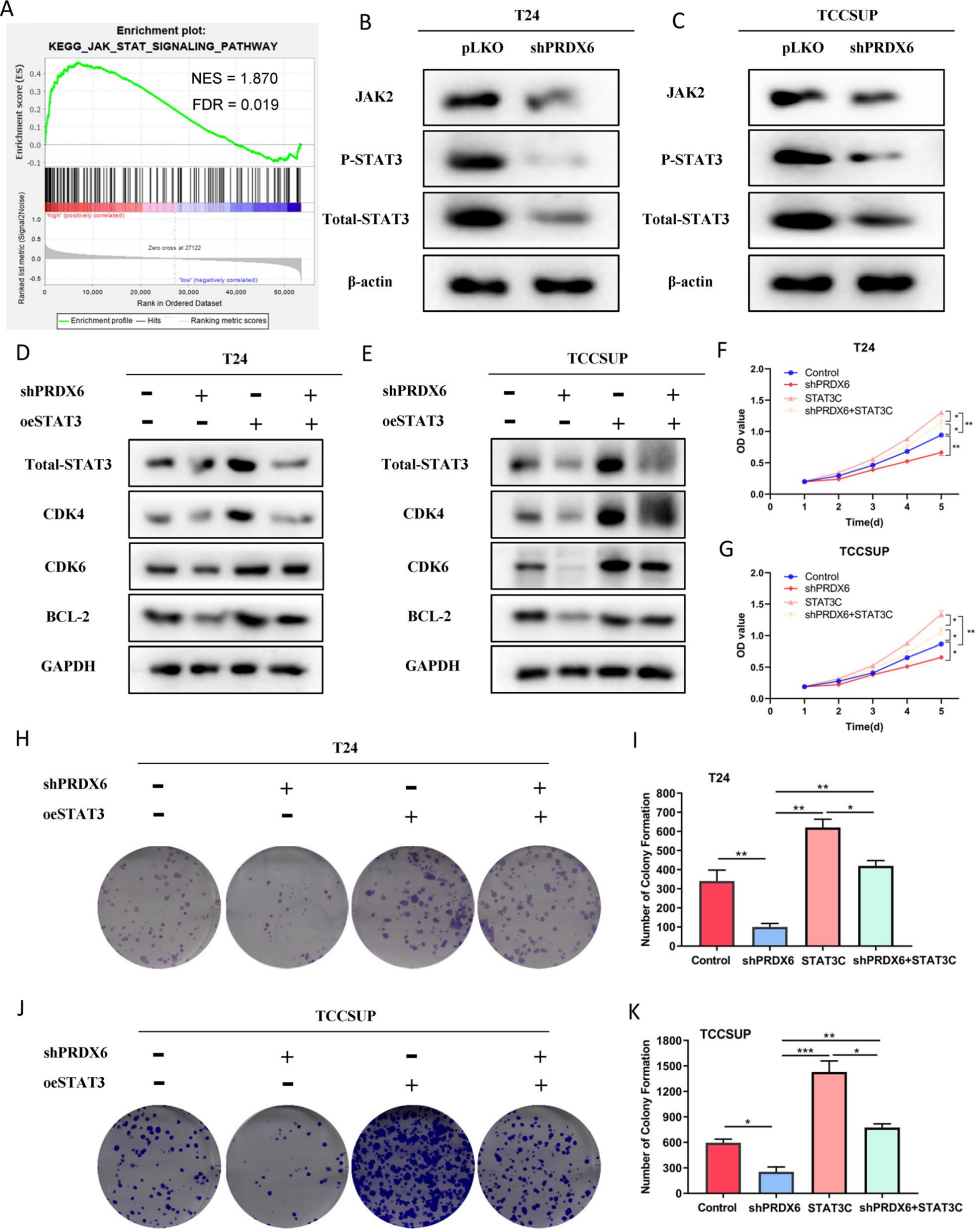
PRDX6 promotes bladder cancer cell proliferation via JAK2-STAT3 pathway. **A** The GSEA plot of JAK/STAT pathway between PRDX6 high and low group. **B**, **C** Western blot was used to test JAK2, p-STAT3 and STAT3 protein both in T24 cells (**B**) and TCCSUP cells (**C**). **D**–**G** To test whether PRDX6 regulate the cell growth via JAK2-STAT3 pathway, western blot assays and CCK8 were performed on T24 cells (**D**, **F**) and TCCSUP cells (**E**, **G**) transfected with control, shPRDX6, oeSTAT3, shPRDX6 + oeSTAT3. **H**–**K** Colony formation were performed on T24 cells (**H**, **I**) and TCCSUP cells (**J**, **K**) transfected with control, shPRDX6, oeSTAT3, shPRDX6 + oeSTAT3. *P < 0.05, **P < 0.01

To investigate the mechanism of PRDX6 regulating cell proliferation signaling, the GSEA analysis was performed, the results showed that JAK-STAT3 pathway was significantly enriched in PRDX high group (Fig. 6E, Fig. 8A). STAT3 plays as an important role in urological related tumors [37], and previous study has confirmed that STAT3 as the upstream signaling of CDK4/6 pathway [38]. Taken together, we speculated that PRDX6 modulates JAK-STAT3 signaling thus regulating proliferation of BLCA cells. Western blot results showed that PRDX6 knockdown decreased JAK2 protein level phosphorylation and total STAT3 protein level in T24 and TCCSUP cells (Fig. 8B, C). Then the decreased protein levels of STAT3, CDK4, CDK6 and BCL-2 induced by shPRDX6 can be reversed through addition of oeSTAT3 (Fig. 8D, E). What’s more, the results of CCK8 (Fig. 8F, G) and colony formation (Fig. 8H, K) demonstrated that the diminished effect of proliferation by knockdown PRDX6 can be reversed by oeSTAT3 in T24 and TCCSUP cells. Therefore, we can conclude that PRDX6 could promote the cell proliferation through regulating JAK2-STAT3 signaling in BLCA.

## Discussion

Accumulating studies demonstrated that PRDXs play vital roles in the progression of cancers, however, a systematic analysis of PRDXs in different cancers is deficiency. Therefore, it is necessary to study the expression, genetic alterations, regulation patterns and potential drugs for diagnosis and treatment of cancers with aberrantly PRDXs expression. The aim of our study was to explore the characteristic features of PRDXs in pan-cancer and their potential roles in BLCA. Increased or decreased levels of PRDXs mRNA expression levels were found among 33 tumor types, such as PRDX1, PRDX2, PRDX4 and PRDX5 increased in several cancer types, while PRDX3 and PRDX6 decreased in there types of kidney cancer (KIRP, KIRC and KICH). Nicolussi et al. [39] reviewed that overexpression of PRDXs were found in several cancers, especially PRDX6. The TCGA dataset showed that mRNA expression levels of PRDX6 increased in BLCA, CESC, ESCA, LIHC, LUAD, LUSC, PRAD, THCA, UCEC, and OV. Interestingly, the expression patterns of PRDX6 in BRCA were contrary in different dataset, such as mRNA expression levels of PRDX6 decreased in TCGA dataset [40], while protein levels increased in breast cancer cells [26], which may be caused by individual difference, race, cancer subtypes.

As for survival analysis, PRDX1, PRDX4 and PRDX6 were mostly associated with poor survival of OS, DSS and PFI, PRDX2 and PRDX3 with favorable survival, while PRDX5 was risky factors for LAML, LIHC, UVM and protective factors for KIRP, LGG, MESO in OS. As we all known, epigenetic aberrations involve the initiation and progression of tumors [41]. Therefore, the genetic alteration and methylation of PRDXs were explored. The results showed that PRDX gene expressions were positively correlated with CNV and negatively with methylation, implying that PRDXs are probably epigenetic-drive genes.

Subsequently, the correlations between PRDXs and immune subtypes, tumor stemness demonstrated that PRDX1, PRDX4, PRDX6 were closely related with type C1, C2 and C6 infiltrates, while tumor stemness scores were positively with all PRDX members. One possible explanation is that PRDXs are differentially expressed in immune responses, For example the *Leishmania donovani* peroxidoxin: LdPxn1 is upregulated during the amastigote stage whereas LdPxn2 increased in the promastigote stage [42]. Moreover, PRDXs are functionally different [43]. Considering that tumor stemness were significantly correlated with drug response, then the relationships between 10 tumor-related pathways and potential drugs were investigated. The results showed that PRDXs were associated with apoptosis and cell cycle pathway. Moreover, analysis of data from PharmacoDB showed high expression levels of PRDX1, PRDX3, PRDX6 were positively correlated with a number of FDA approved EGFR/VEGFR inhibitor drugs, such as Pazopanib, Vandetanib, Lapatinib and Cediranib (*P* < 0.001). These drugs are used to treat tumors such as renal cell carcinoma [44], melanoma, thyroid cancer [45]. These findings suggest that reasonable use of EGF/VEGF inhibitors can effectively treat some cancers with aberrant expression of PRDXs. Besides, Gemcitabine, Doxorubicin, AZD8055 (mTOR inhibitor), GDC-0941 (PI3K inhibitor), Irinotecan (cytotoxic chemotherapy drug), 17-AAG (HSP90 inhibitor), Mitomycin-C (cytotoxic), and temsirolimus drugs were also strongly related with high expression of PRDXs. These drugs regulate cancer by modulating cancer metabolism. Furthermore, findings on genetic alterations and methylation showed that PRDX members had significant positive correlations with CNV and methylation, which indicates that the combination of epigenetic inhibitors and EGF/VEGF inhibitors may be an effective anticancer treatment approach.

Abnormal expression levels of PRDX members are associated with OS, but most previous studies only focused on several members, and systematic analysis of PRDXs in BLCA has not been reported. Therefore, the present study explored the characteristics of PRDXs in BLCA. The results demonstrated that PRDX1, PRDX4, PRDX5 and PRDX6 were significant difference between tumor and normal tissues, which were also validated in HPA database. In addition, KM plot showed that high expression levels of PRDX1, PRDX4 and PRDX6 were associated with poor prognosis, were in consistent with the researches of Soini et al. [46] and Quan et al. [30]. Finally, PRDX6, which had 13% genetic alterations and correlations with CNV, methylation was 0.61 (*P* < 0.01) and -0.32 (*P* < 0.01), was selected to explore the functions of PRDXs in BLCA. T24 and TCCSUP bladder cancer cell lines were used for PRDX6 knockdown and analysis of cell growth and cell cycle. Our results showed that the low expression levels of PRDX6 could inhibit the growth of cancer cells, which were in accord with the results of esophageal carcinoma [47], breast cancer [26]. Subsequently, GSEA analysis was used to explore the potential biological pathways associated with PRDXs in BLCA. The results showed that PRDXs are positively associated with JAK/STAT pathway. Previous literature reported that overexpression peroxiredoxin 4 is a complex interplay of antioxidant and several signaling functions, such as NF-κB-mediated proinflammatory response and JAK/STAT-mediated stress response in fly physiology [48]. Moreover, Yun et al. reported that PRDX6 promotes tumor progress via the JAK2/STAT3 pathway in lung tumor model [49]. Therefore, in order to explore whether PRDX6 functions via JAK/STAT, then we detect the famous modules of JAK/STAT, including JAK2, STAT3 and p-STAT3. The results exhibited that PRDX6 could regulate the cell proliferation through JAK2-STAT3 pathway and is implicated in the process of cell cycle, which were in consistent with the results from lung cancer model [49]. Therefore, PRDX6 is a potential novel target for the treatment of BLCA.

Although the PRDX family members generally exhibit features of oncogenes, the effects on cancer survival and cancer cell proliferation are different. One possible explanation for this variation is that aberrant expression of PRDX family members involve interaction with different factors, such as response elements, transcription factors, tumor suppressors and microRNAs in different human tumors [50, 51].

This study also has some limitations. Although we revealed the significance of PRDXs in the progress of 33 tumors and validate the function of RPDX6 in BLCA, the effects and mechanisms should be confirmed using clinical samples and animal experiments. The specific experiment will be designed in detail in further research.

## Conclusion

In summary, our study provided systematic analyses of PRDXs in pan-cancer, including expression profiles, associations with survival information, genetic alteration, methylation, potential biological pathways and association with drugs. Moreover, experiments in vitro showed that PRDX6 could regulate cell proliferation via JAK2-STAT3 signaling and involve the cell cycle process in BLCA, which may be more valuable for personalized treatment of cancer. In a word, we not only uncover the pivotal roles of the PRDXs in the progression of 33 types of cancer, but validate that knocking down PRDX6 could attenuate the cell growth through JAK2-STAT3 pathway in BLCA.

## Supplementary Information

**Additional file 1: Figure S1.** The barplots of PRDX expression between normal and tumor tissues in different cancer types. (A) PRDX1. (B) PRDX2. (C) RPDX3. (D) PRDX4. (E) PRDX5. (F) PRDX6. **Figure S2.** The survival analysis of PRDXs in different cancers. (A)DFI, (B)DSS, (C)PFI. **Figure S3.** PRDXs mutation in pan-cancer based on cBioportal website. (A) Oncoprinter of PRDXs in different cancer types. (B) Amino acid mutation of PRDXs. The important mutation sites that predicted as damaging in both AhpC-TSA and 1-cysPrx_C algorithms in the functional and structural importance of the protein sequence position. **Figure S4.** The correlations among expression levels of PRDXs with diagnostic values, tumor stages. (A) ROC curves, (B) barplots between PRDXs and tumor stages. **Table S1**. The associations of PRDXs and mRNAsi. **Table S2**. The univariate cox and multivariate cox of PRDXs and clinical characteristics. **Table S3**. The associations between target drugs and PRDX expressions.

## Data Availability

The data of 33 types of cancers could be download from TCGA dataset (https://portal.gdc.cancer.gov/) and cBioportal (https://www.cbioportal.org/). The experimental data generated or analyzed during this paper are included in this original data.

## Abbreviations

PRDXs: Peroxiredoxins
ROS: Reactive oxygen species
OS: Overall survival
DFI: Disease free interval
PFI: Progression free interval
DSS: Disease specific survival
TCGA: The Cancer Genome Atlas
BLCA: Bladder cancer
ESCA: Esophageal carcinoma
KICH: Kidney chromophobe
KIRC: Kidney renal clear cell carcinoma
KIRP: Kidney renal papillary cell carcinoma
LIHC: Liver hepatocellular carcinoma
LUAD: Lung adenocarcinoma
LUSC: Lung squamous cell carcinoma
DLBC: Lymphoid neoplasm diffuse large B-cell lymphoma
MESO: Mesothelioma
MISC: Miscellaneous
OV: Ovarian serous cystadenocarcinoma
PAAD: Pancreatic adenocarcinoma
PCPG: Pheochromocytoma and paraganglioma
PRAD: Prostate adenocarcinoma
READ: Rectum adenocarcinoma
SARC: Sarcoma
SKCM: Skin cutaneous melanoma
STAD: Stomach adenocarcinoma
TGCT: Testicular germ cell tumors
THYM: Thymoma
THCA: Thyroid carcinoma
UCS: Uterine carcinosarcoma
UCEC: Uterine corpus endometrial carcinoma
UVM: Uveal melanoma
LGG: Brain lower grade glioma
BRCA: Breast invasive carcinoma
CNV: Copy number variation
EMT: Epithelial-to-mesenchymal transition
RTK: Receptor tyrosine kinases
AR: Androgen receptor
HPA: Human Protein Atlas
KM: Kaplan Meier
GSEA: Gene set enrichment
shPRDX6: Shorthair pin PRDX6
oeSTAT: Overexpression STAT3
CCK8: Cell Counting Kit-8

## References

[1] Nelson KJ, Knutson ST, Soito L, Klomsiri C, Poole LB, Fetrow JS (2011). Analysis of the peroxiredoxin family: using active-site structure and sequence information for global classification and residue analysis. Proteins.

[2] Neumann CA, Fang Q (2007). Are peroxiredoxins tumor suppressors?. Curr Opin Pharmacol.

[3] Lee YJ (2020). Knockout mouse models for peroxiredoxins. Antioxidants.

[4] Rhee SG, Kang SW, Chang TS, Jeong W, Kim K (2001). Peroxiredoxin, a novel family of peroxidases. IUBMB Life.

[5] Hall A, Karplus PA, Poole LB (2009). Typical 2-Cys peroxiredoxins–structures, mechanisms and functions. FEBS J.

[6] Soito L, Williamson C, Knutson ST, Fetrow JS, Poole LB, Nelson KJ (2011). PREX: PeroxiRedoxin classification indEX, a database of subfamily assignments across the diverse peroxiredoxin family. Nucleic Acids Res..

[7] Kumar Y, Biswas T, Thacker G, Kanaujiya JK, Kumar S, Shukla A, Khan K, Sanyal S, Chattopadhyay N, Bandyopadhyay A, Trivedi AK (2018). BMP signaling-driven osteogenesis is critically dependent on Prdx-1 expression-mediated maintenance of chondrocyte prehypetrophy. Free Radic Biol Med.

[8] Huh JY, Kim Y, Jeong J, Park J, Kim I, Huh KH, Kim YS, Woo HA, Rhee SG, Lee KJ, Ha H (2012). Peroxiredoxin 3 is a key molecule regulating adipocyte oxidative stress, mitochondrial biogenesis, and adipokine expression. Antioxid Redox Signal.

[9] Jeong SJ, Kim S, Park JG, Jung IH, Lee MN, Jeon S, Kweon HY, Yu DY, Lee SH, Jang Y, Kang SW, Han KH, Miller YI, Park YM, Cheong C, Choi JH, Oh GT (2018). Prdx1 (peroxiredoxin 1) deficiency reduces cholesterol efflux via impaired macrophage lipophagic flux. Autophagy.

[10] Kim SU, Park YH, Kim JM, Sun HN, Song IS, Huang SM, Lee SH, Chae JI, Hong S, Sik Choi S, Choi SC, Lee TH, Kang SW, Rhee SG, Chang KT, Lee SH, Yu DY, Lee DS (2014). Dominant role of peroxiredoxin/JNK axis in stemness regulation during neurogenesis from embryonic stem cells. Stem Cells.

[11] Fujii J, Ikeda Y (2002). Advances in our understanding of peroxiredoxin, a multifunctional, mammalian redox protein. Redox Rep.

[12] Wang X, Phelan SA, Petros C, Taylor EF, Ledinski G, Jurgens G, Forsman-Semb K, Paigen B (2004). Peroxiredoxin 6 deficiency and atherosclerosis susceptibility in mice: significance of genetic background for assessing atherosclerosis. Atherosclerosis.

[13] Lv WP, Li MX, Wang L (2017). Peroxiredoxin 1 inhibits lipopolysaccharide-induced oxidative stress in lung tissue by regulating P38/JNK signaling pathway. Eur Rev Med Pharmacol Sci.

[14] Ding C, Fan X, Wu G (2017). Peroxiredoxin 1 - an antioxidant enzyme in cancer. J Cell Mol Med.

[15] Kim JH, Lee JM, Lee HN, Kim EK, Ha B, Ahn SM, Jang HH, Lee SY (2012). RNA-binding properties and RNA chaperone activity of human peroxiredoxin 1. Biochem Biophys Res Commun.

[16] Lee DJ, Kang DH, Choi M, Choi YJ, Lee JY, Park JH, Park YJ, Lee KW, Kang SW (2013). Peroxiredoxin-2 represses melanoma metastasis by increasing E-Cadherin/beta-Catenin complexes in adherens junctions. Cancer Res.

[17] Hu JX, Gao Q, Li L (2013). Peroxiredoxin 3 is a novel marker for cell proliferation in cervical cancer. Biomed Rep.

[18] Kim YS, Lee HL, Lee KB, Park JH, Chung WY, Lee KS, Sheen SS, Park KJ, Hwang SC (2011). Nuclear factor E2-related factor 2 dependent overexpression of sulfiredoxin and peroxiredoxin III in human lung cancer. Korean J Intern Med.

[19] Choi JH, Kim TN, Kim S, Baek SH, Kim JH, Lee SR, Kim JR (2002). Overexpression of mitochondrial thioredoxin reductase and peroxiredoxin III in hepatocellular carcinomas. Anticancer Res.

[20] Karihtala P, Mantyniemi A, Kang SW, Kinnula VL, Soini Y (2003). Peroxiredoxins in breast carcinoma. Clin Cancer Res.

[21] Jiang H, Wu L, Mishra M, Chawsheen HA, Wei Q (2014). Expression of peroxiredoxin 1 and 4 promotes human lung cancer malignancy. Am J Cancer Res.

[22] Chang KP, Yu JS, Chien KY, Lee CW, Liang Y, Liao CT, Yen TC, Lee LY, Huang LL, Liu SC, Chang YS, Chi LM (2011). Identification of PRDX4 and P4HA2 as metastasis-associated proteins in oral cavity squamous cell carcinoma by comparative tissue proteomics of microdissected specimens using iTRAQ technology. J Proteome Res.

[23] Pritchard C, Mecham B, Dumpit R, Coleman I, Bhattacharjee M, Chen Q, Sikes RA, Nelson PS (2009). Conserved gene expression programs integrate mammalian prostate development and tumorigenesis. Cancer Res.

[24] Karihtala P, Kauppila S, Soini Y, Arja Jukkola V (2011). Oxidative stress and counteracting mechanisms in hormone receptor positive, triple-negative and basal-like breast carcinomas. BMC Cancer.

[25] Pylvas M, Puistola U, Kauppila S, Soini Y, Karihtala P (2010). Oxidative stress-induced antioxidant enzyme expression is an early phenomenon in ovarian carcinogenesis. Eur J Cancer.

[26] Chang XZ, Li DQ, Hou YF, Wu J, Lu JS, Di GH, Jin W, Ou ZL, Shen ZZ, Shao ZM (2007). Identification of the functional role of peroxiredoxin 6 in the progression of breast cancer. Breast Cancer Res..

[27] Raatikainen S, Aaaltomaa S, Karja V, Soini Y (2015). Increased peroxiredoxin 6 expression predicts biochemical recurrence in prostate cancer patients after radical prostatectomy. Anticancer Res.

[28] Kalinina EV, Berezov TT, Shtil AA, Chernov NN, Glazunova VA, Novichkova MD, Nurmuradov NK (2012). Expression of peroxiredoxin 1, 2, 3, and 6 genes in cancer cells during drug resistance formation. Bull Exp Biol Med.

[29] Pak JH, Choi WH, Lee HM, Joo WD, Kim JH, Kim YT, Kim YM, Nam JH (2011). Peroxiredoxin 6 overexpression attenuates cisplatin-induced apoptosis in human ovarian cancer cells. Cancer Invest.

[30] Quan C, Cha EJ, Lee HL, Han KH, Lee KM, Kim WJ (2006). Enhanced expression of peroxiredoxin I and VI correlates with development, recurrence and progression of human bladder cancer. J Urol.

[31] Thorsson V, Gibbs DL, Brown SD, Wolf D, Bortone DS, Ou Yang TH, Porta-Pardo E, Gao GF, Plaisier CL, Eddy JA, Ziv E, Culhane AC, Paull EO, Sivakumar IKA, Gentles AJ, Malhotra R, Farshidfar F, Colaprico A, Parker JS, Mose LE, Vo NS, Liu J, Liu Y, Rader J, Dhankani V, Reynolds SM, Bowlby R, Califano A, Cherniack AD, Anastassiou D, Bedognetti D, Mokrab Y, Newman AM, Rao A, Chen K, Krasnitz A, Hu H, Malta TM, Noushmehr H, Pedamallu CS, Bullman S, Ojesina AI, Lamb A, Zhou W, Shen H, Choueiri TK, Weinstein JN, Guinney J, Saltz J, Holt RA, Rabkin CS, Lazar AJ, Serody JS, Demicco EG, Disis ML, Vincent BG, Shmulevich I, Cancer Genome Atlas Research N (2018). The Immune Landscape of Cancer. Immunity..

[32] Malta TM, Sokolov A, Gentles AJ, Burzykowski T, Poisson L, Weinstein JN, Kaminska B, Huelsken J, Omberg L, Gevaert O, Colaprico A, Czerwinska P, Mazurek S, Mishra L, Heyn H, Krasnitz A, Godwin AK, Lazar AJ, Stuart JM, Hoadley KA, Laird PW, Noushmehr H, Wiznerowicz M, Cancer Genome Atlas Research N (2018). Machine Learning Identifies Stemness features associated with oncogenic dedifferentiation. Cell..

[33] Gao L, Meng J, Zhang M, Fan S, Gao S, Wang X, Liang C (2020). Expression and prognostic values of the roof plate-specific spondin family in bladder cancer. DNA Cell Biol.

[34] Gu J, Zhang Y, Han Z, Gao L, Cui J, Sun Y, Niu Y, You B, Huang CP, Chang C, Wang X, Yeh S (2020). Targeting the ERbeta/Angiopoietin-2/Tie-2 signaling-mediated angiogenesis with the FDA-approved anti-estrogen Faslodex to increase the Sunitinib sensitivity in RCC. Cell Death Dis..

[35] Tamborero D, Rubio-Perez C, Muinos F, Sabarinathan R, Piulats JM, Muntasell A, Dienstmann R, Lopez-Bigas N, Gonzalez-Perez A (2018). A pan-cancer landscape of interactions between solid tumors and infiltrating immune cell populations. Clin Cancer Res.

[36] Smirnov P, Kofia V, Maru A, Freeman M, Ho C, El-Hachem N, Adam GA, Ba-Alawi W, Safikhani Z, Haibe-Kains B (2018). PharmacoDB: an integrative database for mining in vitro anticancer drug screening studies. Nucleic Acids Res.

[37] Hindupur SV, Schmid SC, Koch JA, Youssef A, Baur EM, Wang D, Horn T, Slotta-Huspenina J, Gschwend JE, Holm PS, Nawroth R (2020). STAT3/5 inhibitors suppress proliferation in bladder cancer and enhance oncolytic adenovirus therapy. Int J Mol Sci..

[38] Aboushousha T, Hammam O, Aref A, Kamel A, Badawy M, Abdel Hamid A (2020). Tissue profile of CDK4 and STAT3 as possible innovative therapeutic targets in urinary bladder cancer. Asian Pac J Cancer Prev.

[39] Nicolussi A, D'Inzeo S, Capalbo C, Giannini G, Coppa A (2017). The role of peroxiredoxins in cancer. Mol Clin Oncol.

[40] Wang G, Zhong WC, Bi YH, Tao SY, Zhu H, Zhu HX, Xu AM (2019). The prognosis of peroxiredoxin family in breast cancer. Cancer Manag Res.

[41] Villanueva L, Alvarez-Errico D, Esteller M (2020). The Contribution of epigenetics to cancer immunotherapy. Trends Immunol.

[42] Barr SD, Gedamu L (2001). Cloning and characterization of three differentially expressed peroxidoxin genes from Leishmania chagasi. Evidence for an enzymatic detoxification of hydroxyl radicals. J Biol Chem..

[43] Barr SD, Gedamu L (2003). Role of peroxidoxins in Leishmania chagasi survival evidence of an enzymatic defense against nitrosative stress. J Biol Chem..

[44] Li W, Feng C, Di W, Hong S, Chen H, Ejaz M, Yang Y, Xu TR (2020). Clinical use of vascular endothelial growth factor receptor inhibitors for the treatment of renal cell carcinoma. Eur J Med. Chem..

[45] Lee CS, Baek J, Han SY (2017). The role of kinase modulators in cellular senescence for use in cancer treatment. Molecules.

[46] Soini Y, Haapasaari KM, Vaarala MH, Turpeenniemi-Hujanen T, Karja V, Karihtala P (2011). 8-hydroxydeguanosine and nitrotyrosine are prognostic factors in urinary bladder carcinoma. Int J Clin Exp Pathol.

[47] He Y, Xu W, Xiao Y, Pan L, Chen G, Tang Y, Zhou J, Wu J, Zhu W, Zhang S, Cao J (2018). Overexpression of peroxiredoxin 6 (PRDX6) promotes the aggressive phenotypes of esophageal squamous cell carcinoma. J Cancer.

[48] Radyuk SN, Klichko VI, Michalak K, Orr WC (2013). The effect of peroxiredoxin 4 on fly physiology is a complex interplay of antioxidant and signaling functions. FASEB J.

[49] Yun HM, Park KR, Park MH, Kim DH, Jo MR, Kim JY, Kim EC, Yoon DY, Han SB, Hong JT (2015). PRDX6 promotes tumor development via the JAK2/STAT3 pathway in a urethane-induced lung tumor model. Free Radic Biol Med.

[50] Ismail T, Kim Y, Lee H, Lee DS, Lee HS (2019). Interplay between mitochondrial peroxiredoxins and ROS in cancer development and progression. Int J Mol Sci..

[51] Kwee JK (2014). A paradoxical chemoresistance and tumor suppressive role of antioxidant in solid cancer cells: a strange case of Dr. Jekyll and Mr. Hyde. BioMed research international.

