# Supplementary material for: Integrative analysis the characterization of peroxiredoxins in pan-cancer
Source: Cancer Cell Int. 2021 Jul 10;21:366. doi: 10.1186/s12935-021-02064-x (PMC8272277; doi:10.1186/s12935-021-02064-x)
Supplement: Supplementary file 1 — Additional file 1: Figure S1. The barplots of PRDX expression between normal and tumor tissues in different cancer types. (A) PRDX1. (B) PRDX2. (C) RPDX3. (D) PRDX4. (E) PRDX5. (F) PRDX6. Figure S2. The survival analysis of PRDXs in different cancers. (A)DFI, (B)DSS, (C)PFI. Figure S3. PRDXs mutation in pan-cancer based on cBioportal website. (A) Oncoprinter of PRDXs in different cancer types. (B) Amino acid mutation of PRDXs. The important mutation sites that predicted as damaging in both AhpC-TSA and 1-cysPrx_C algorithms in the functional and structural importance of the protein sequence position. Figure S4. The correlations among expression levels of PRDXs with diagnostic values, tumor stages. (A) ROC curves, (B) barplots between PRDXs and tumor stages. Table S1. The associations of PRDXs and mRNAsi. Table S2. The univariate cox and multivariate cox of PRDXs and clinical characteristics. Table S3. The associations between target drugs and PRDX expressions. [file 12935_2021_2064_MOESM1_ESM.pdf]

Figure.S1

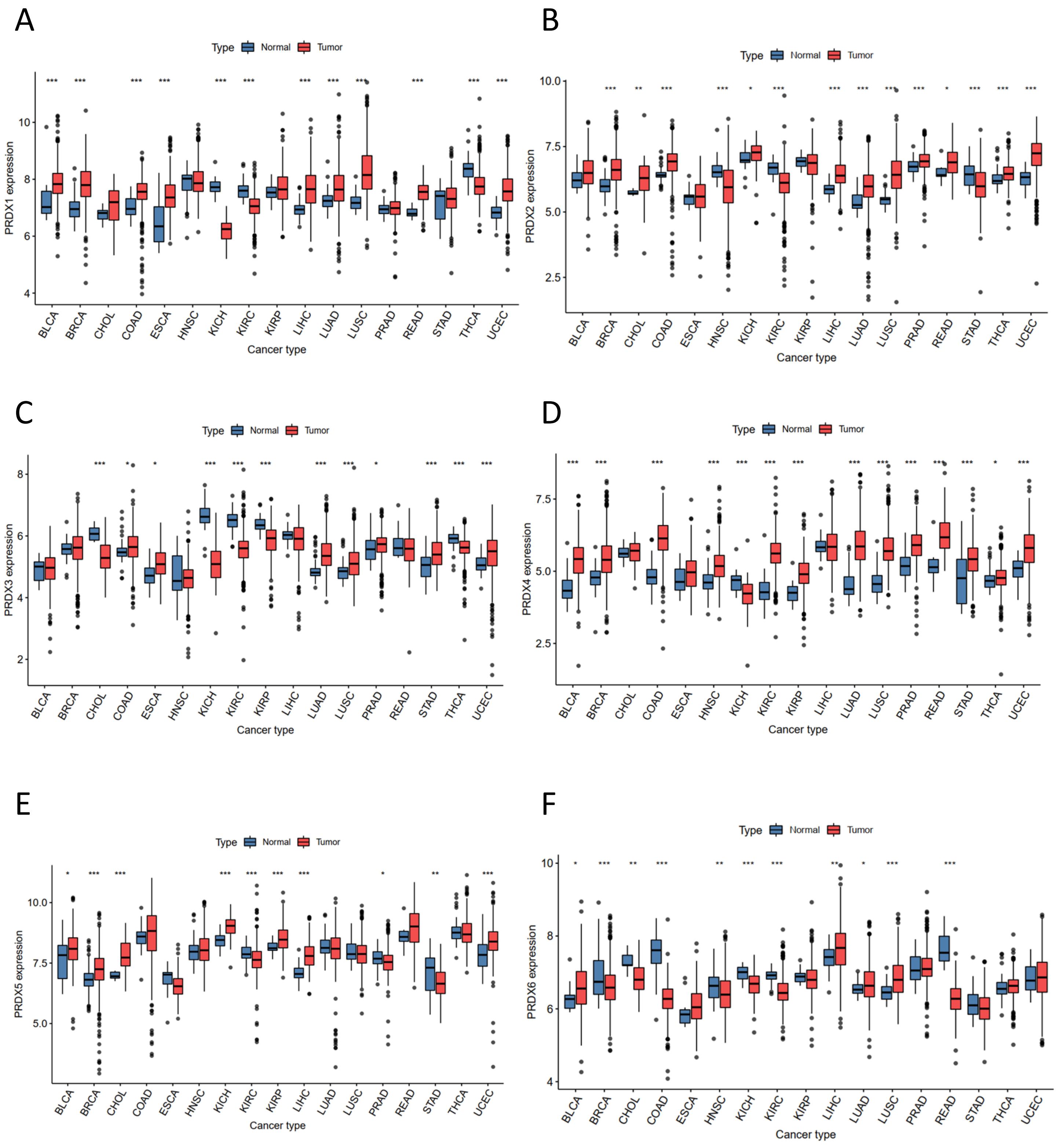

A
Type Normal Tumor
PRDX1 expression
Cancer type
B
Type Normal Tumor
PRDX2 expression
Cancer type
C
Type Normal Tumor
PRDX3 expression
Cancer type
D
Type Normal Tumor
PRDX4 expression
Cancer type
E
Type Normal Tumor
PRDX5 expression
Cancer type
F
Type Normal Tumor
PRDX6 expression
Cancer type
BLCA BRCA CHOL COAD ESCA HNSC KICH KIRC KIRP LIHC LUAD LUSC PRAD READ STAD THCA UCEC

Figure.S2

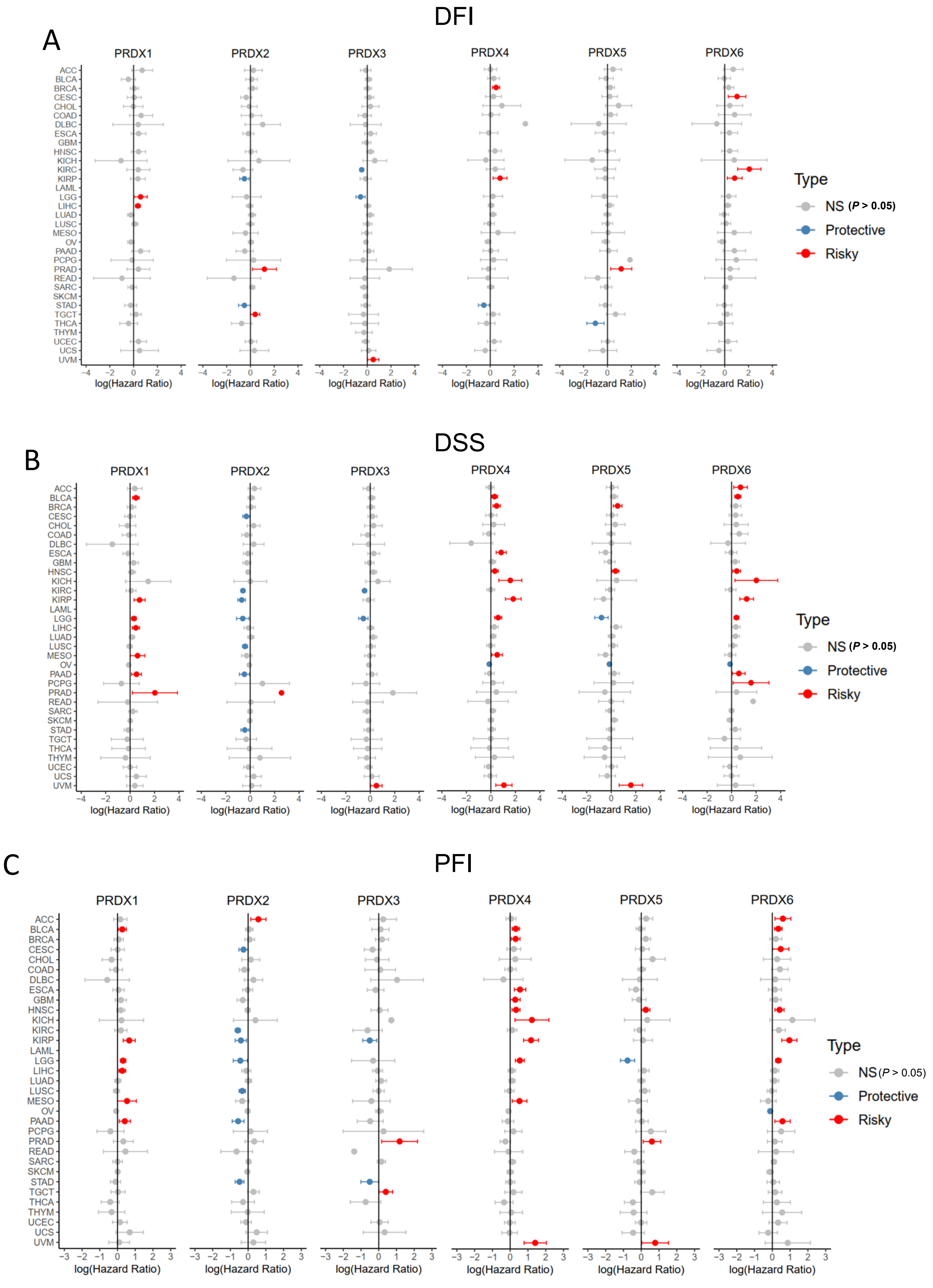
DFI
A
DSS
B
PFI
C
PRDX1
PRDX2
PRDX3
PRDX4
PRDX5
PRDX6
ACC
BLCA
BRCA
CESC
CHOL
COAD
DLBC
ESCA
GBM
HNSC
KICH
KIRC
KIRP
LAML
LGG
LIHC
LUAD
LUSC
MESO
OV
PAAD
PCPG
PRAD
READ
SARC
SKCM
STAD
TGCT
THCA
THYM
UCEC
UCS
UVM
log(Hazard Ratio)
Type
NS (P > 0.05)
Protective
Risky

Figure.S3

A

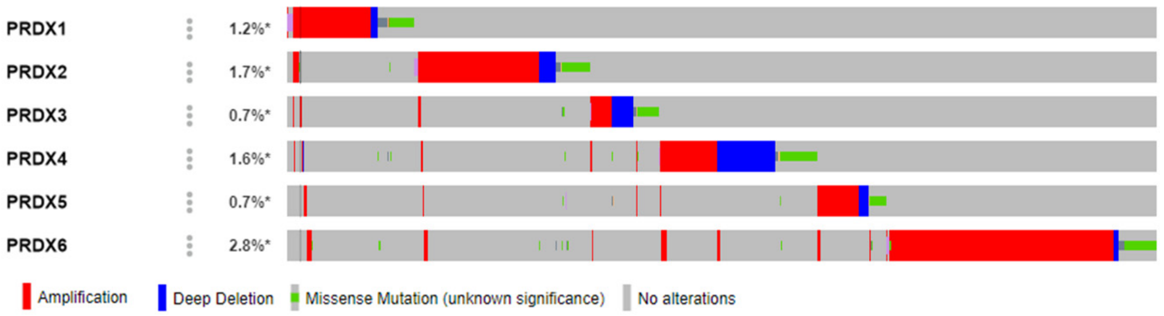
PRDX1
1.2%*
PRDX2
1.7%*
PRDX3
0.7%*
PRDX4
1.6%*
PRDX5
0.7%*
PRDX6
2.8%*
Amplification
Deep Deletion
Missense Mutation (unknown significance)
No alterations

B

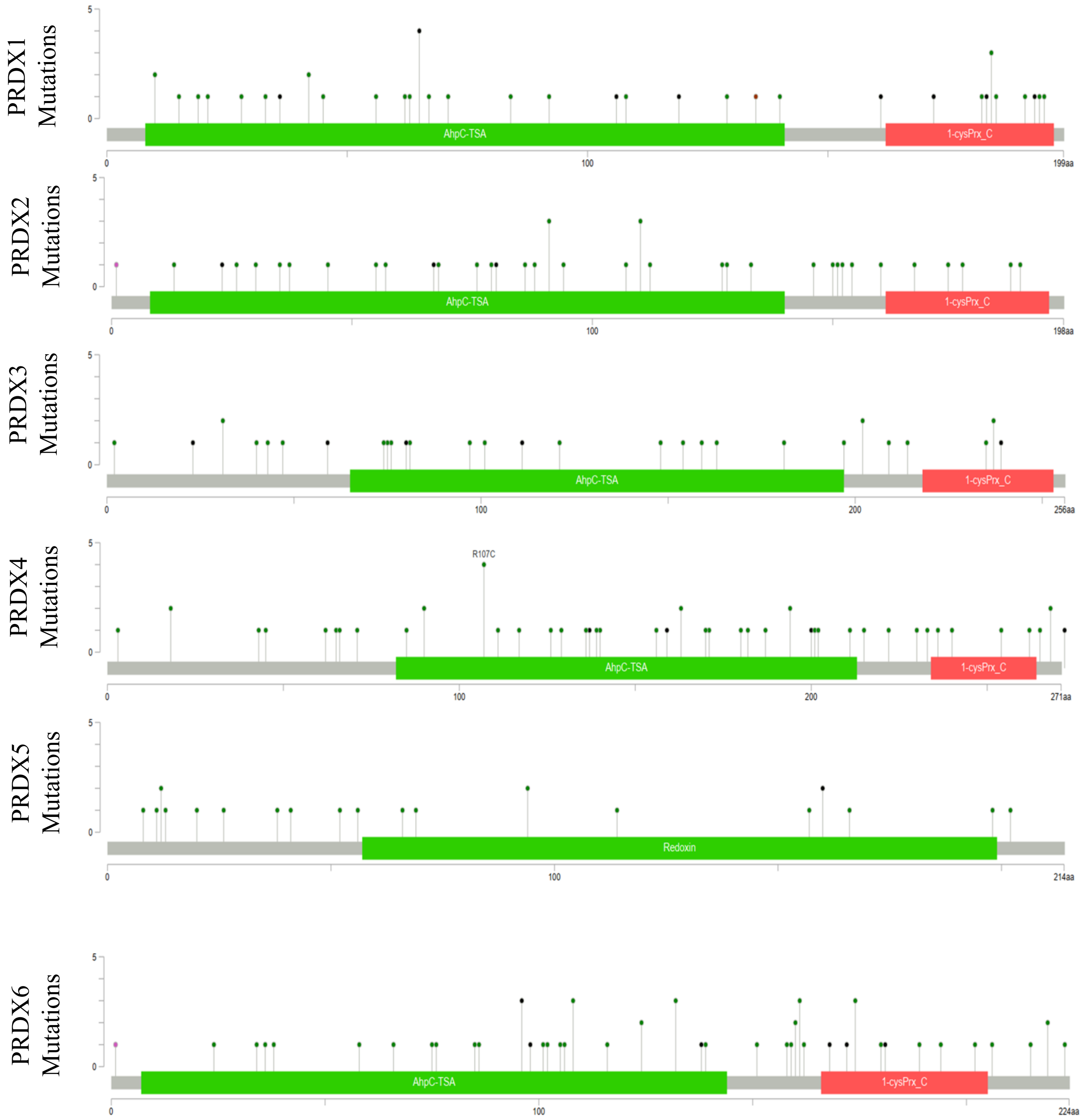
PRDX1 Mutations
AhpC-TSA
1-cysPrx_C
199aa
PRDX2 Mutations
AhpC-TSA
1-cysPrx_C
198aa
PRDX3 Mutations
AhpC-TSA
1-cysPrx_C
256aa
PRDX4 Mutations
R107C
AhpC-TSA
1-cysPrx_C
271aa
PRDX5 Mutations
Redoxin
214aa
PRDX6 Mutations
AhpC-TSA
1-cysPrx_C
224aa

Figure.S4

A

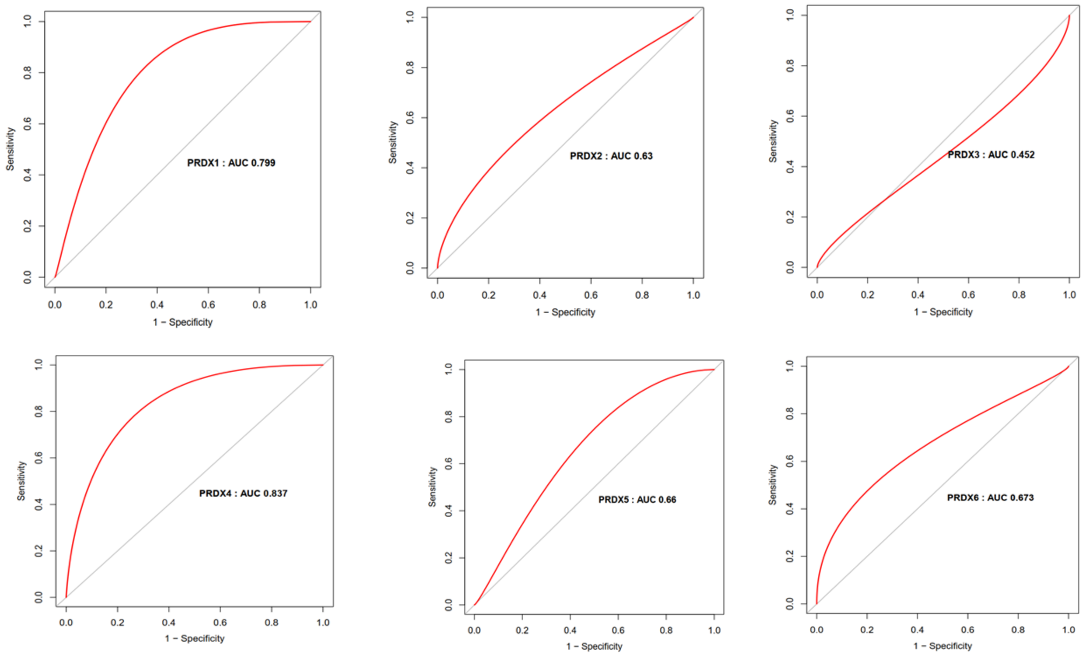
PRDX1 : AUC 0.799
PRDX2 : AUC 0.63
PRDX3 : AUC 0.452
PRDX4 : AUC 0.837
PRDX5 : AUC 0.66
PRDX6 : AUC 0.673
Sensitivity
1 − Specificity

B

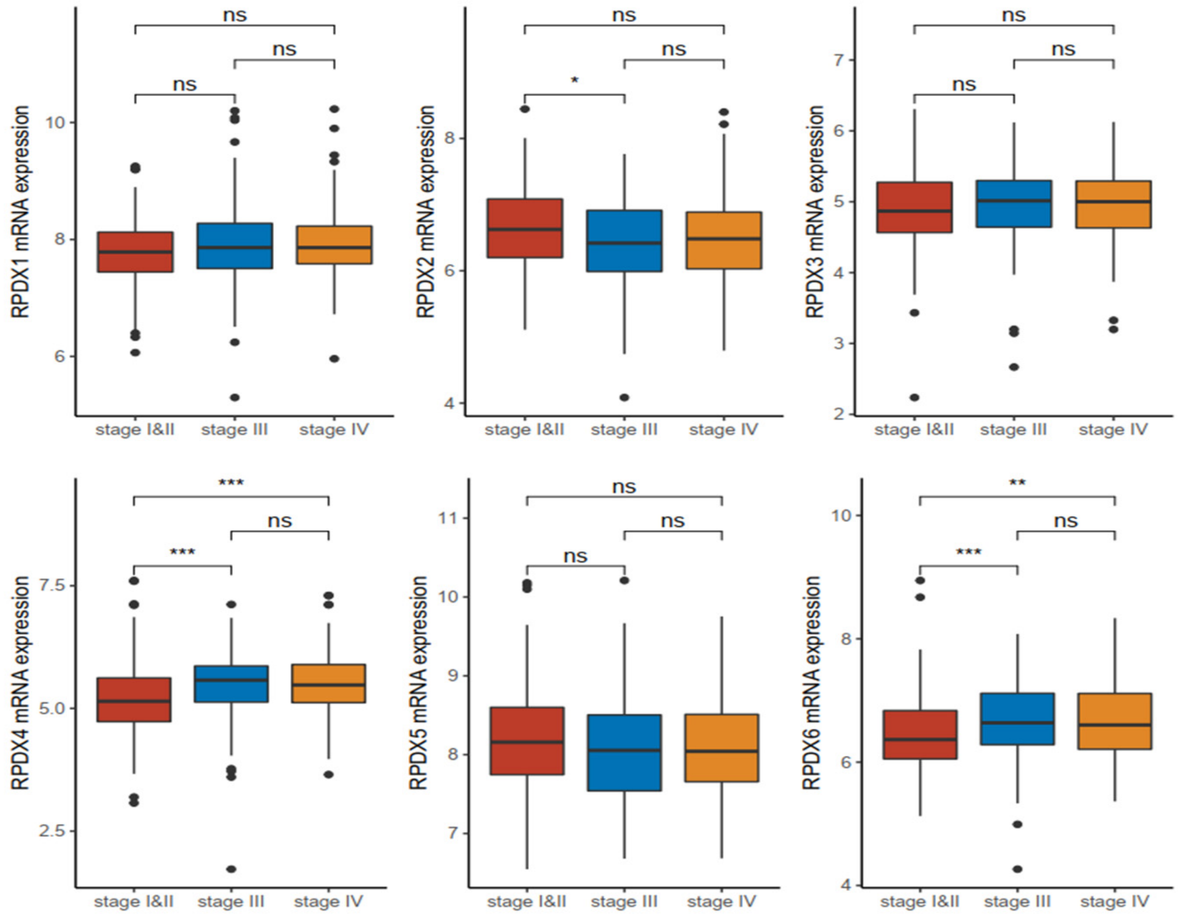
RPDX1 mRNA expression
RPDX2 mRNA expression
RPDX3 mRNA expression
RPDX4 mRNA expression
RPDX5 mRNA expression
RPDX6 mRNA expression
stage I&II
stage III
stage IV
ns
*
**
***

Table S1 The associations of PRDXs and mRNAsi.

CancerType	PRDX1		PRDX2		PRDX3	
	cor	pvalue	cor	pvalue	cor	pvalue
TGCT	0.662515	0	-0.06596	0.414386	0.26808	0.000775
UCEC	0.499293	0	0.320639	6.16E-14	0.246003	1.18E-08
BRCA	0.456143	0	0.236001	3.99E-15	0.062662	0.038969
HNSC	0.447386	0	0.454016	0	0.190253	2.07E-05
LUSC	0.432584	0	0.458464	0	0.205984	4.91E-06
UCS	0.431032	0.001013	0.459945	0.000418	0.225632	0.094539
LUAD	0.417209	0	0.214372	1.12E-06	0.460025	0
COAD	0.390515	0	0.357645	1.13E-14	0.416368	0
KIRP	0.388799	1.55E-11	0.271295	3.82E-06	0.408927	8.98E-13
OV	0.382456	1.76E-10	0.351494	5.47E-09	0.368605	8.60E-10
LIHC	0.368428	3.32E-13	0.153647	0.003074	-0.04513	0.386505
STAD	0.363988	1.21E-12	0.383337	4.92E-14	0.385884	3.07E-14
SARC	0.315967	2.53E-07	0.125838	0.043493	0.186771	0.002633
READ	0.309497	9.13E-05	0.305394	0.000114	0.3868	7.54E-07
CESC	0.302641	9.00E-08	0.279571	8.46E-07	0.013018	0.821357
MESO	0.287042	0.007555	-0.05522	0.612938	0.232228	0.031648
BLCA	0.276518	1.83E-08	0.404675	0	0.166558	0.000789
KIRC	0.267753	8.05E-10	0.214567	9.68E-07	0.363213	8.25E-18
PAAD	0.265948	0.000823	0.263264	0.000933	0.125365	0.118835
ESCA	0.257846	0.001031	0.356243	4.39E-06	0.448271	4.11E-09
CHOL	0.250965	0.139583	0.098842	0.564956	0.198713	0.244315
GBM	0.216541	0.005156	0.484728	4.26E-11	0.00322	0.967132
PRAD	0.192063	1.86E-05	0.345228	4.19E-15	0.433857	0
THCA	0.171614	0.000109	0.332477	2.28E-14	0.394367	0
SKCM	0.165132	0.000325	-0.05564	0.228042	0.25464	2.35E-08
LAML	0.041478	0.619966	0.021973	0.792835	0.416194	2.52E-07
DLBC	0.03528	0.811404	-0.01465	0.921231	0.310139	0.032367
ACC	-0.08689	0.4486	0.475512	1.39E-05	0.227937	0.044946
KICH	-0.10375	0.413622	0.498397	3.55E-05	0.078571	0.536229
UVM	-0.14534	0.197928	0.071683	0.526709	0.215026	0.055594
LGG	-0.40949	0	0.397624	0	0.19773	5.44E-06
PCPG	-0.41279	9.69E-09	0.292609	6.57E-05	0.197628	0.007575
THYM	-0.57007	0	0.666764	0	0.163018	0.076528

(continue)

CancerType	PRDX4		PRDX5		PRDX6	
	cor	pvalue	cor	pvalue	cor	pvalue
TGCT	0.359035	5.21E-06	-0.05548	0.492538	0.004921	0.951502
UCEC	0.273262	2.11E-10	0.041268	0.344275	0.36993	0
BRCA	0.461305	0	0.062224	0.040359	0.357279	0
HNSC	0.090357	0.044313	0.101004	0.024515	0.376125	0
LUSC	0.003724	0.934707	0.016492	0.716766	0.445858	0
UCS	-0.01784	0.896022	0.241012	0.073706	0.085988	0.527581
LUAD	0.315692	4.05E-13	0.009767	0.825946	0.439484	0
COAD	0.313762	1.92E-11	0.144268	0.002383	0.26202	2.55E-08
KIRP	0.235281	6.53E-05	0.336008	7.89E-09	0.347797	2.18E-09
OV	0.273163	7.27E-06	0.211506	0.000556	0.280531	4.02E-06
LIHC	0.099751	0.055261	0.366381	4.62E-13	0.291637	1.28E-08
STAD	0.361655	1.74E-12	0.221967	2.13E-05	0.318356	7.13E-10
SARC	0.161406	0.009467	0.134931	0.030325	0.354139	6.22E-09
READ	0.175294	0.028729	0.127477	0.112713	0.245329	0.002077
CESC	0.129904	0.02379	-0.01952	0.7349	0.274216	1.38E-06
MESO	0.057069	0.60108	0.276513	0.010163	0.338271	0.001529
BLCA	0.094718	0.057172	-0.07398	0.13764	0.096825	0.05184
KIRC	0.168548	0.000126	0.2471	1.53E-08	0.327502	3.48E-14
PAAD	0.139856	0.081641	0.28816	0.00028	0.436598	1.73E-08
ESCA	0.293367	0.000177	0.187774	0.017536	0.236262	0.002693
CHOL	0.050193	0.770682	0.058172	0.735368	0.380952	0.022539
GBM	-0.02262	0.772193	0.203355	0.008692	-0.33106	1.48E-05
PRAD	0.383037	0	-0.01697	0.70715	0.118887	0.008298
THCA	-0.07716	0.083228	0.2027	4.61E-06	0.253386	8.71E-09
SKCM	-0.07963	0.084297	0.090325	0.050128	-0.02843	0.538035
LAML	0.369887	5.48E-06	-0.27407	0.000886	0.334168	4.42E-05
DLBC	-0.014	0.924734	0.052215	0.72377	0.348676	0.015583
ACC	0.099736	0.384186	0.25085	0.026996	0.204315	0.072854
KICH	-0.01914	0.880476	0.071291	0.574741	0.262134	0.036689
UVM	0.227496	0.042618	0.023019	0.839105	0.105767	0.349711
LGG	0.039735	0.364468	0.275996	1.61E-10	-0.55083	0
PCPG	-0.2549	0.000536	0.237951	0.001253	-0.07755	0.297788
THYM	-0.38582	1.71E-05	0.043249	0.64003	0.296589	0.001105

Table S2 The univariate cox and multivariate cox of PRDXs and clinical characteristics.

**Characterics**	**Unicox**			**Multicox**		
	**HR**	**95%CI**	***P***	**HR**	**95%CI**	***P***
age	1.033	1.017-1.049	**<0.001**	1.031	1.014-1.048	**0.00031**
gender	0.897	0.648-1.241	0.512	0.897	0.644-1.251	0.522
stage	2.228	1.539-3.227	**<0.001**	2.066	1.408-3.032	**<0.001**
grade	0.344	0.085-1.388	0.133	0.749	0.178-3.16	0.695
PRDX1	1.445	1.147-1.82	**0.001**	1.158	0.896-1.499	0.262
PRDX2	0.984	0.791-1.224	0.884	1.046	0.835-1.309	0.697
PRDX3	0.969	0.736-1.276	0.821	0.998	0.736-1.355	0.991
PRDX4	1.184	0.953-1.469	0.126	0.939	0.734-1.201	0.615
PRDX5	1.024	0.805-1.304	0.845	1.143	0.887-1.473	0.301
PRDX6	1.394	1.11-1.75	**0.004**	1.275	0.979-1.659	**0.071**

Table S3 The associations between target drugs and PRDXs expression levels.

Drug	PRDX1	PRDX2	PRDX3	PRDX4	PRDX5	PRDX6
Vandetanib	0.194647	-0.0182	0.563382	0.339325	0.114883	0.438373
Gemcitabine	0.149144	0.070469	0.213025	-0.05886	-0.19928	0.051208
AZD8055	0.147519	0.150818	0.084566	0.031925	-0.04212	0
Z-LLNle-CHO	0.131679	0	-0.06794	0.019206	-0.03942	0.090389
GDC-0941	0.131137	0.065384	0.317817	0.104295	-0.04263	0.010344
Doxorubicin	0.128033	0.110062	0.179063	-0.10524	-0.17022	0.478478
Irinotecan	0.11119	0.094764	0.184622	-0.12484	-0.18223	0.041502
17-AAG	0.107565	0	0.180897	-0.04648	0.054854	0.091082
lapatinib	0.087885	0.036537	0.102143	-0.14497	0.120357	0.086827
Mitomycin-C	0.08725	0.184876	0.104634	-0.07691	-0.12918	0.025398
temsirolimus	0.082543	0.145909	0.107624	0.264633	-0.05428	0.363514
Crizotinib	0.081912	0.095706	0.319006	0.263822	-0.07995	0.527947
Pazopanib	0.053164	0	0.200807	0.20384	-0.0525	0.478478
BIBR-1532	0.050339	0.182783	0.051799	0.012032	-0.02248	0
GNF-2	0.021132	0	-0.04187	-0.05813	-0.09106	0.200791
FTI-277	0.018747	0	-0.0439	-0.00509	0.082744	0.033056
bortezomib	0	0.167132	0	0	0	0
TAE684	-0.01805	0.089642	-0.12224	0.154983	0.094301	0.077648
OSI-906	-0.03159	0.074328	-0.06358	0.089355	0.076518	-0.05009
salermide	-0.04906	0.168997	-0.03464	0.032716	0	0
cediranib	-0.10654	0.04304	0.051591	0.118544	-0.02912	-0.08283
BIX-01294	-0.11153	0.18406	-0.10008	-0.09922	-0.09112	-0.17917
staurosporine	-0.12849	0.005329	0.019075	0.174245	-0.01122	-0.06783
Topotecan	-0.16783	0.160954	0.182319	-0.08754	-0.11819	-0.05245
ML312	-0.18256	0.105382	-0.07989	0.178524	0.01736	-0.02497

Freescience Editorial Team

Certificate of English Editing

Paper Title

Integrative analysis the characterization of peroxiredoxins in pan-cancer

Authors

Lei Gao1†, Jialin Meng2†, Chuang Yue3†, Xingyu Wu3, Quanxin Su3, Hao Wu3, Ze Zhang3, Qinzhou Yu2, Shenglin Gao3*, Song Fan2*, Li Zuo3*

This certificate is issued as a confirmation that the paper mentioned above has been proofread and edited for language clarity and grammar by professional editors of our company.

We guarantee that the original message was not distorted, and that the paper is understandable and free of errors assuming that the changes and suggestions given are accepted, and text is not altered without our knowledge

Date of Editing: 06-11-2021
